# Activation of AhR with nuclear IKKα regulates cancer stem-like properties in the occurrence of radioresistance

**DOI:** 10.1038/s41419-018-0542-9

**Published:** 2018-04-30

**Authors:** Bin Yan, Shuang Liu, Ying Shi, Na Liu, Ling Chen, Xiang Wang, Desheng Xiao, Xiaoli Liu, Chao Mao, Yiqun Jiang, Weiwei Lai, Xing Xin, Can-E Tang, Dixian Luo, Tan Tan, Jiantao Jia, Yating Liu, Rui Yang, Jun Huang, Hu Zhou, Yan Cheng, Ya Cao, Weishi Yu, Kathrin Muegge, Yongguang Tao

**Affiliations:** 1 Institute of Medical Sciences, Xiangya Hospital, Central South University, 87 Xiangya Road, Changsha, 410008 Hunan China; 2 Key Laboratory of Carcinogenesis and Cancer Invasion, Ministry of Education, Xiangya Hospital, Central South University, 87 Xiangya Road, Changsha, 410008 Hunan China; 3 Cancer Research Institute, Central South University, 110 Xiangya Road, Changsha, 410078 Hunan China; 4 Department of Thoracic Surgery, Second Xiangya Hospital, Central South University, Changsha, China; 5 Department of Pathology, Xiangya Hospital, Central South University, Changsha, 410008 Hunan China; 6 National and Local Joint Engineering Laboratory of High-throughput Molecular Diagnosis Technology, Translational Medicine Institute, the First People’s Hospital of Chenzhou, University of South China, 102 Luojiajing Road, Chenzhou, 423000 Hunan China; 7 Department of Pathophysiology, Changzhi Medical College, Changzhi, Shanxi China; 8 Department of Neurosugery, Xiangya Hospital, Central South University, 87 Xiangya Road, Changsha, 410078 Hunan China; 9 Shanghai Institute of Material Medica, Chinese Academy of Sciences (CAS), 555 Zu Chongzhi Road, Zhangjiang Hi-Tech Park, 201203 Shanghai, China; 10 Department of Pharmacology, School of Pharmaceutical Sciences, Central South University, Changsha, 410078 Hunan China; 11 Cipher Gene (Beijing) Co. Ltd., 100089 Beijing, China; 12 Mouse Cancer Genetics Program, National Cancer Institute, Basic Science Program, Leidos Biomedical Research, Inc., Frederick National Laboratory for Cancer Research, Frederick, MD 21702 USA

## Abstract

Most cancer patients receive radiotherapy in the course of their disease and the occurrence of radioresistance is associated with poor prognosis. The molecular pathways that drive enhanced tumorigenic potential during the development of radioresistance are poorly understood. Here, we demonstrate that aryl hydrocarbon receptor (AhR) plays a vital role in the maintenance of cancer stem-like properties. AhR promotes the cancer stem-like phenotype and drives metastasis by directly targeting the promoters of ‘stemness’ genes, such as the ATP-binding cassette sub-family G member 2 (ABCG2) gene. Moreover, the radioresistant sublines display high levels of oncometabolites including α-ketoglutarate, and treatment of cancer cells with α-ketoglutarate enhances their stem-like properties in an AhR activation-dependent manner. IKKα directly activates stemness-related genes through an interaction with AhR as a bone fide chromatin modifier. Thus, AhR is functionally linked with cancer stem-like properties, and it drives tumorigenesis in the occurrence of radioresistance.

## Introduction

The aryl hydrocarbon receptor (AhR), a ligand-operated transcription factor, is a xenosensor traditionally associated with xenobiotic metabolism^1^. AhR facilitates tumor progression, disease tolerance defense, intestinal immunity, and B-cell proliferation^2–5^. Interestingly, AhR influences the major stages of tumorigenesis, and studies of aggressive tumors and tumor cell lines have shown increased levels of AhR protein and constitutive nuclear localization in cancer tissue, whereas in normal tissues AhR is mainly inactive and resides in the cytoplasm^6,7^.

The activation of nuclear factor (NF)-κB leads to a protumorigenic inflammatory microenvironment, and the IκB-kinase (IKK) complex, which consists of two catalytic subunits, IKKα and IKKβ, and a regulatory subunit, IKKγ, tightly regulates the NF-κB pathway^8,9^. Whereas, in most malignancies, the classical IKKβ/IKKγ-dependent NF-κB activation controls key functions for tumor initiation, promotion, and progression in tumors^10^. The role of IKKα is more complex in noncanonical NF-κB pathway^11,12^. Depending on the type of malignancy, IKKα can provide both tumor-promoting and tumor-suppressive mechanisms that are in most instances cell autonomous^13^.

Radiotherapy, using ionizing radiation, is a commonly applied procedure for the treatment of cancers including lung cancer (LC) and nasopharyngeal carcinoma (NPC). Even though the technology of radiotherapy, including the quality of the equipment and the precision of targeting, has greatly improved over the last decades, residual tumor tissues after irradiation and relapse due to radioresistant cancer cells remain a major challenge. The small radioresistant tumor subpopulation, known as “cancer stem cells” (CSCs), possesses specific molecular properties that protects it against radiation-induced damage and plays a critical role in tissue invasion and metastasis^14–16^. Several markers are known to characterize CSCs, including CD133, CD44, ATP-binding cassette sub-family G member 2 (ABCG2, also named as CD338), and epithelial cell adhesion molecule (Epcam, also named as CD326), stemness-related transcription factors Nanog, Octamer binding transcription factor 4 (Oct4), Krüppel-like factor 4 (KLF4), and aldehyde dehydrogenase (ALDH) activity^15,17–19^. While several of these genes promote the stemness of CSCs, their exact roles in radioresistance have not been fully elucidated.

Accumulating evidence supports the existence of CSCs such as those derived from irradiation-resistant cells that possess the capacity to self-renew and to differentiate into bulk tumor cells^20^. In this study, we report that AhR is functionally linked with cancer stem-like properties, and it drives tumorigenesis in the occurrence of radioresistance.

## Results

### Radioresistant sublines of cancer cells display increased tumorigenic, stem-like and metastasis properties

**Fig. 1:**
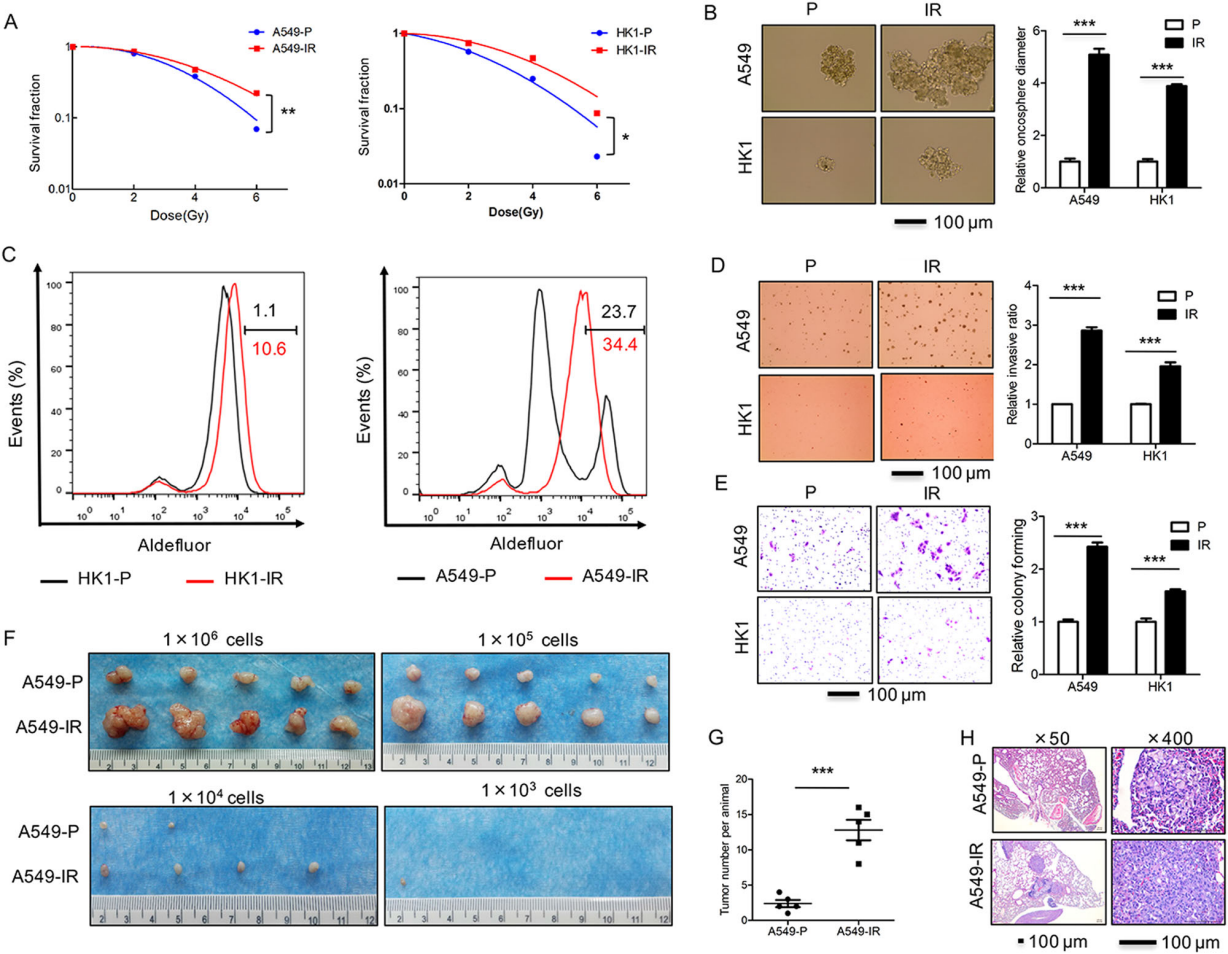
Radioresistant sublines of cancer cells display increased tumorigenic, stem-like and metastasis properties. **a** Radiobiological survival colony formation assay comparing irradiation resistant A549-IR, HK1-IR, and H358-IR vs. parental (P) lines (*n* = 3). **b** A sphere growth assay is shown in A549-IR and HK1-IR cells as compared to P lines (left) and the results are summarized in the bar graph (right, *n* = 3). **c** The ALDH activity in A549-IR and HK1-IR cells as compared to P cells by aldefluor assay (*n* = 3). **d** A representative soft-agar assay in A549-IR and HK1-IR cells as compared to P cells is shown (left) and the results are summarized in the bar graph (right, *n* = 3). **e** A representative trans-well assay in A549-IR and HK1-IR cells as compared to P cells is shown (left) and the results are summarized in the bar graph (right, *n* = 3). **f** Dilution assay in tumor formation is shown after subcutaneous injection of A549-IR and A549-P cells into SCID mice with different amounts of cells as indicated (*n* = 5 for each group), arrow indicated tumor in the group of 1×10^3^ cells. **g**, **h** Tumor number measured for A549-P and A549-IR sublines that were injected into the tail vein of SCID mice. Animals (*n* = 6 for each group) were euthanized and the development of lung metastases was assessed macroscopically (**g**) or by microscope using paraffin-embedded sections stained with H&E (**h**); *p *<* 0.05, **p *<* 0.01, ***p *<* 0.001

As a tool to identify markers of radioresistance in cancer cells, we used ionizing radiation resistant (IR) sublines generated from epithelial cancer cell lines HK1, A549, and H358. The first line is derived from the tissues of NPC, whereas the latter two are lung adenocarcinoma. Cell cultures were treated with multiple fractions of 4 Gy of X-rays to a total dose of more than 80 Gy. The radiobiological clonogenic assay indicated enhanced survival in irradiation resistant (IR) sublines compared to the non-irradiated parental (P) cell lines. A significant increase in the survival of IR cells compared to P cells was observed at all given doses (Fig. 1a, and Supplementary Figure S1A). We observed that both A549-IR and HK1-IR cells exhibited more stem-like properties, such as the capacity of sphere growth (Fig. 1b) and aldefluor assay in ALDH activity (Fig. 1c), compared to P cells. Furthermore, all IR sublines in A549-IR, HK1-IR, and H358-IR cells showed greater survival in the soft-agar colony assay and enhanced in vitro invasion ability compared to P cell lines (Fig. 1d, e and Supplementary Figure S1B).

To investigate in vivo tumor formation, we injected the P and IR sublines subcutaneously into Balb/C immune-deficient mice (2 × 10^6^ cells/mouse). After 2 months, we found significantly larger tumors from A549-IR cells as compared to the parental A54-P cells; moreover, limiting dilution assays were further performed to determine the frequency of radioresistant sublines in A549-IR and P lines from xenografts (Fig. 1f), significantly A549-IR efficiently formed large subcutaneous tumors. To further examine the properties of metastasis, we directly injected the tumor cells (1 × 10^6^ cells/mouse) into the tail vein of Balb/C nu/nu mice. All A549-IR recipient mice (5/5) exhibited increased numbers of metastasis compared to mice that received the P cells (Fig. 1g, h and Supplementary Figure S1C-D). Taken together, our results demonstrate that the IR sublines show greater tumorigenic, stem-like and metastatic potential compared to their P lines.

### The radioresistant sublines of cancer cells display elevated stem-like genes including ABCG2

**Fig. 2:**
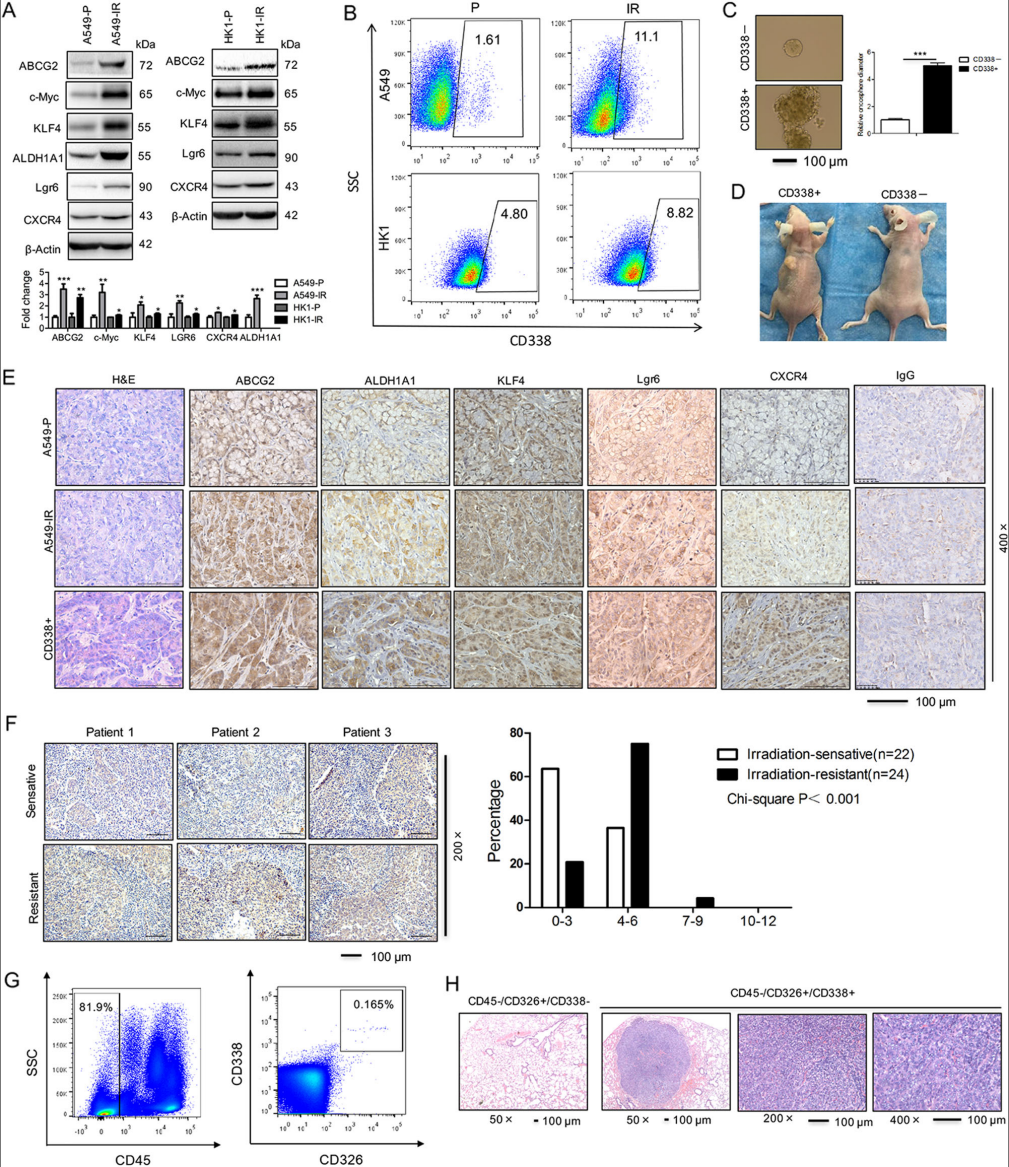
The radioresistant sublines of cancer cells display elevated stem-like genes including ABCG2. **a** Western blot for detection of the expression of stemness-related genes as indicated in A549-P and A549-IR sublines (left), HK1-P and HK1-IR cells (right) (*n* = 3). The mean values of western blot quantification are shown at the bottom. **b** Representative images of flow cytometry analysis for detection of CD338-positive cells in A549-IR (up) and HK1-IR (bottom) sublines (*n* = 3). **c** Representative images of CD338-positive and -negative cells that were seeded in tumorspheric culture medium (*n* = 3). **d** Nude mice are shown after injection of CD338-positive and -negative cells from A549-IR cells. **e** H&E staining (left) and immunohistochemical analysis to determine the expression of stemness-related genes as indicated in tumor samples generated in nude mice. **f** Immunohistochemical analysis of ABCG2 in three images of radiosensitive and radioresistant NPC tissues (left) and anti-ABCG2 staining intensity was quantified in three microscopic fields for each tissues section to determine the ABCG2 expression level (right). **g** Representative images of flow cytometry analysis for CD45−/CD326+/CD338+ cells derived from five lung cancer patients. **h** Animals (*n* = 5 for each group) were euthanized and the development of lung metastases derived from CD45−/CD326+/CD338+ cells from lung cancer patients was assessed by microscope using paraffin-embedded sections stained with H&E; *p *<* 0.05, **p *<* 0.01, ***p *<* 0.001

Side populations (SP) in cells may exhibit stem cell-like characteristics. Flow cytometry analysis indicated that the percentage of SP cells was greater in HK1-IR cells (10.6%) compared to P cells (1.57%) (Supplementary Figure S2A). IR cells in A549 and H358 expressed elevated mRNA of genes associated with a stem-like phenotype including ABCG2, c-Myc, KLF4, ALDH1A1, Lgr6, and CXCR4 (Supplementary Figure S2B). Moreover, the protein level of ABCG2, c-Myc, KLF4, ALDH1A1, Lgr6, and CXCR4 was elevated in A549-IR cells as compared to P cells (left panel of Fig. 2a), whereas the protein level of ABCG2, c-Myc, KLF4, Lgr6, and CXCR4 was elevated in HK1-IR cells as compared to P cells (right panel of Fig. 2a), similar findings were seen in H358-IR cells compared to P cells (Supplementary Figure S2C). In addition, the percentage of CD338-positive cells, as measured by flow cytometry analysis, was higher in A549-IR and HK1-IR cells as in their respective P lines (Fig. 2b). We observed that CD338-positive cells exhibited more stem-like properties in the capacity for sphere growth (Fig. 2c). As few as 1000 CD338-positive A549-IR cells that were subcutaneously injected into Balb/C immune-deficient mice resulted in tumor formation (2/5), compared to no tumor formation (0/5) after injection of CD338 negative cells (A presentative image is shown in Fig. 2d). The expression of CSC markers ABCG2, ALDH1A1, KLF4, and LGR6 was readily detected in biopsies of xenograft tumors after injection of IR cells and after injection of CD338-positive cells into mice compared to P cells (Fig. 2e). Moreover, we examined ABCG2 protein levels in NPC tissues by immunohistochemistry analysis; ABCG2 expression was significantly increased in biopsies of radioresistant NPCs as compared to radiosensitive NPC tissues (Fig. 2f). Finally, we sorted ABCG2 cells derived from five patients with lung adenocarcinoma after permission. To get more specific ABCG2-positive tumor-initiating cells from LC patients, we sorted CD45−/CD326+/CD338+ cells from 10.0 ml of blood cells from LC patients (Fig. 2g); then we injected the cells into the tail vein of Balb/C nu/nu mice for 4 months. Interestingly, all CD45−/CD326+/CD338+ cells recipient mice (5/5) exhibited tumor formation in the lung compared to mice that received the CD45−/CD326−/CD338− cells (0/5) (Fig. 2h and Supplementary Figure S2D). Taken together, our data show an association of radiation resistance with elevated stem-like gene signature and enhanced tumorigenic potential.

### Stem-like genes are directly contributed by AhR in radioresistant sublines

**Fig. 3:**
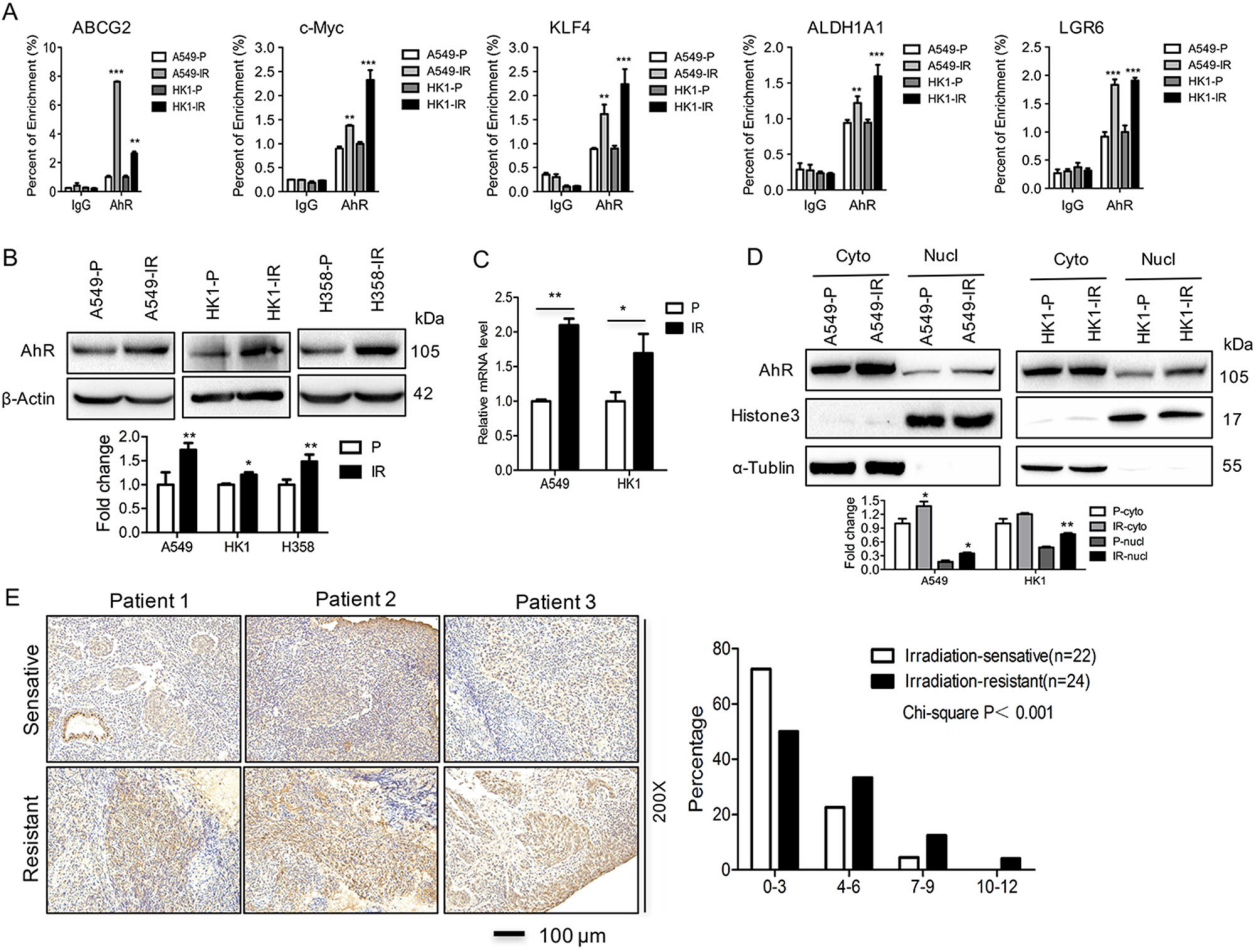
Stem-like genes are directly contributed by AhR in radioresistant sublines. **a** ChIP analysis in A549-IR and HK1-IR sublines and P lines was performed to detect AhR binding at stemness-related genes as indicated (*n* = 4–6). The mean values of western blot quantification are shown at the bottom. **b** Western blot was used to detect AhR expression in radioresistant sublines as indicated (*n* = 3). **c** RT-PCR was used to detect the CYP1A1 mRNA level in A549-IR and HK1-IR sublines and P lines (*n* = 3). **d** Nuclear and cytoplasmic fraction of A549-IR and HK1-IR sublines and P lines showed elevated AhR in the nucleas (*n* = 3). The mean values of western blot quantification are shown at the bottom. **e** Immunohistochemical analysis of AhR in three images of radiosensitive and radioresistant NPC tissues (left) and anti-AhR staining intensity was quantified in three microscopic fields for each tissue section to determine the AhR expression level (right)

To understand more about the regulation of stemness genes, we searched for a common molecular pathway in IR sublines. The UCSC genome browser (www.genome.ucsc.edu) reveals that the promoter regions of stemness marker genes contain AhR binding sites. ChIP analysis demonstrated that AhR was recruited to the promoters of several stemness genes including ABCG2, c-Myc, KLF4, ALDH1A1, and Lgr6 in P cell lines A549 and HK1, suggesting a possible regulatory role of this transcription factor in the expression of stemness genes (Fig. 3a). Notably, the enrichment of AhR at the promoter regions was increased in radioresistant cells lines A549-IR and HK1-IR that showed also higher expression of those stemness-related genes (Fig. 3a). Interestingly, not only AhR promoter occupancy but also the total AhR protein expression levels were consistently elevated in radioresistant sublines compared to P lines (Fig. 3b), whereas the mRNA level of AhR remained the same in radioresistant sublines compared to P lines (data not shown). Moreover, AhR expression was increased in A549-IR and CD338-positive A549-IR and CD338+ biopsies from xenograft tumors (Supplementary Figure S3A); meanwhile, the mRNA level of CYP1A1, a bona fide target gene of AhR, was increased in A549-IR and HK1-IR cells (Fig. 3c), indicating that higher activation of AhR signaling pathway exists in radioresistant sublines.

Since AhR can be found in the cytoplasm and in the nucleus, we confirmed that the nuclear fraction of AhR was indeed increased in A549-IR and HK1-IR cells as an active form (Fig. 3d). Immunofluorescence staining (Supplementary Figure S3B) together with the high content imaging system (Supplementary Figure S3C-E) confirmed that the nuclear localization of AhR increased in the HK1-IR and A549-IR sublines as compared to P cells. We examined the AhR protein levels in control (NP) and NPC tissues by immunohistochemistry analysis. While low levels of AhR protein were detectable in NP tissue, AhR was greatly increased in NPCs, and the degree of expression was associated with expression of the Epstein–Barr virus (EBV)-encoded RNA (Supplementary Figure S4A-B). Furthermore, AhR expression was significantly increased in biopsies of radioresistant NPCs as compared to radiosensitive NPC tissues (Fig. 3e). Also, the expression of AhR protein was elevated in NPC tissues of patients with metastasis compared to those with non-metastasis (Supplementary Figure S4C, D).

### Knockdown of AhR decreases the stemness signature

**Fig. 4:**
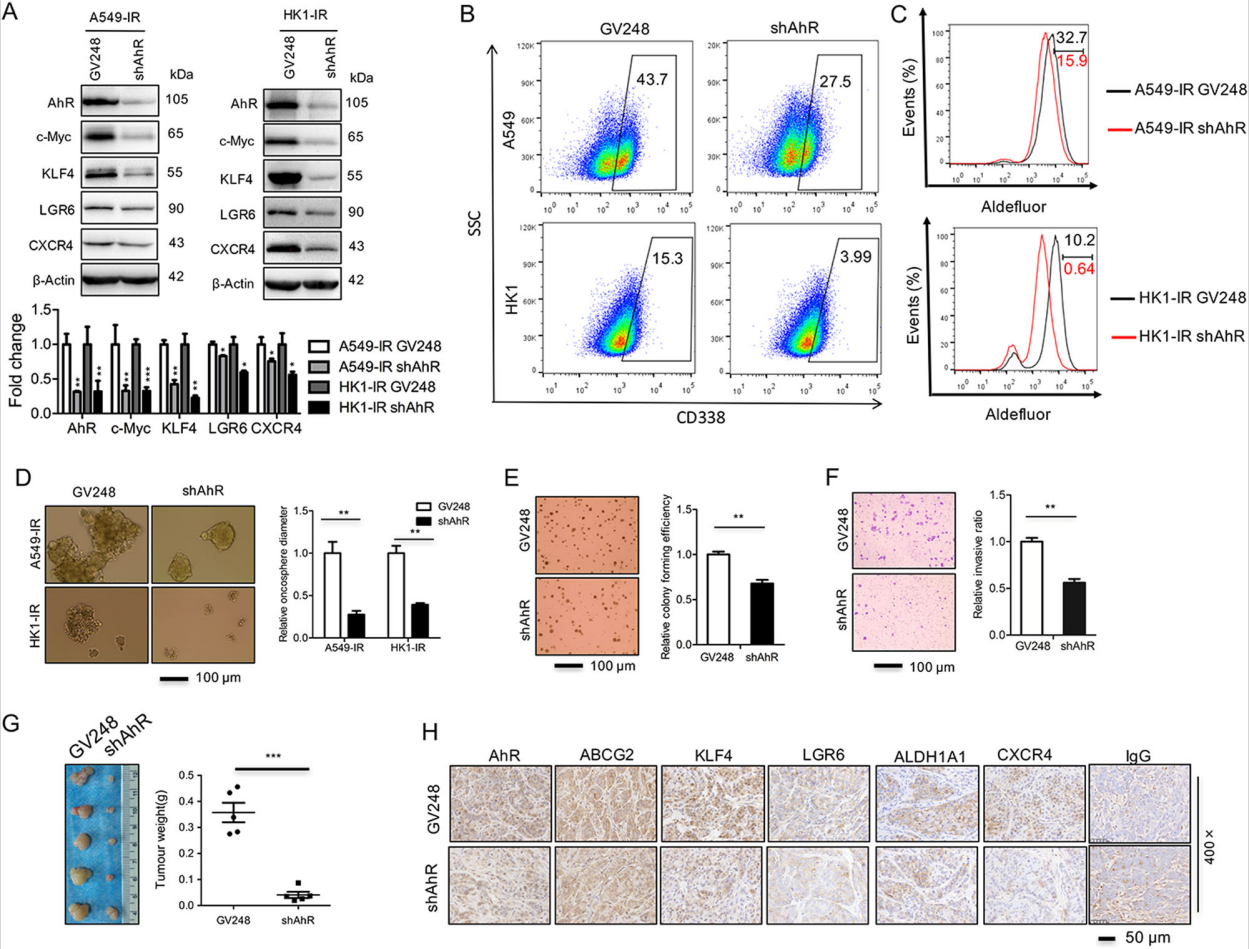
Knockdown of AhR decreases stem-like properties. **a** AhR and stemness protein levels were detected by Western analysis after depletion of AhR in A549-IR (left) and HK1-IR (right) sublines. The mean values of western blot quantification are shown at the bottom. **b** Representative images of flow cytometry analysis for detection of CD338-positive cells in A549-IR (up) and HK1-IR (bottom) sublines after depletion of AhR (*n* = 3). **c** The ALDH activity in A549-IR (up) and HK1-IR (bottom) cells in the depletion of AhR by aldefluor assay (*n* = 3). **d** Representative images of knockdown of AhR in A549-IR and HK1-IR cells that were seeded in tumorspheric culture medium (left) and results are shown as bar graph (right) (*n* = 3). **e** Soft-agar assay is shown as a representative experiment after depletion of AhR in A549-IR cells (left) and quantified in the bar graph (right) (*n* = 4). **f** Migration and invasion is shown as a representative experiment (left) and quantified in the bar graph (right) (*n* = 3). **g** Both tumor volume (left) and tumor weight (right) were measured in A549-IR sublines after depletion of AhR. **h** IHC was performed using antibodies against AhR, ABCG2, KLF4, Lgr6, ALDH1A1, and CXCR4 in xenograft tissues as indicated; *p *<* 0.05, **p *<* 0.01, ***p *<* 0.001

To validate the physiological role of AhR in the radioresistant sublines, we used four different AhR-targeting shRNAs to knock down AhR expression and observed that shAhR-2 consistently achieved over 95% knockdown efficiency (Supplementary Figure S5A). Therefore, unless specified otherwise, shAhR-2 was used in the subsequent studies. As expect, knockdown of AhR decreased the expression of CYP1A1 and several stemness signature genes including ABCG2, c-Myc, KLF4, Lgr6, ALDH1A1, and CXCR4 at mRNA level in A549-IR and HK1-IR cells (Supplementary Figure S5B). Obviously, the knockdown of AhR decreased the protein level of stem-like genes including c-Myc, KLF4, LGR6, and CXCR4 in A549-IR and HK1-IR cells (Fig. 4a). FACS assay showed that knockdown of AhR lead to a decrease of CD338+ expression in A549-IR and HK1-IR cells (Fig. 4b). In the aldefluor assay, ALDH activity decreased after depletion of AhR in A549-IR and HK1-IR cells (Fig. 4c); A549-IR and HK1-IR cells depleted of AhR exhibited less stem-like properties in sphere growth (Fig. 4d), indicating that AhR contributes to stem-like properties in the radiation sublines.

Moreover, knockdown of AhR resulted in significantly reduced growth of A549-IR and HK1-IR cells as well as P cells in culture (Supplementary Figure S5C), and impaired the colony formation of HK1-P, HK1-IR and A549-IR cells in a plate colony formation assay (Supplementary Figure S5D-E). Furthermore, knockdown of AhR in A549-IR cells led to a decreased activity for colony formation in a soft-agar assay (Fig. 4e) and invasion assay (Fig. 4f). Finally, knockdown of AhR resulted in significantly reduced tumor growth and malignant characteristics of A549-IR cells in nude mice (Fig. 4g and Supplementary Figure S5F); the expression of stemness signature genes including ABCG2, c-Myc, KLF4, Lgr6, ALDH1A1, and CXCR4 were readily decreased in biopsies of xenograft tumors after knockdown of AhR in A549-IR cells (Fig. 4h). Taken together, these findings indicate a critical role of AhR in the maintenance of stem-like properties of the IR sublines.

### Activation of AhR signaling pathway is linked to stem-like properties in radioresistant sublines

**Fig. 5:**
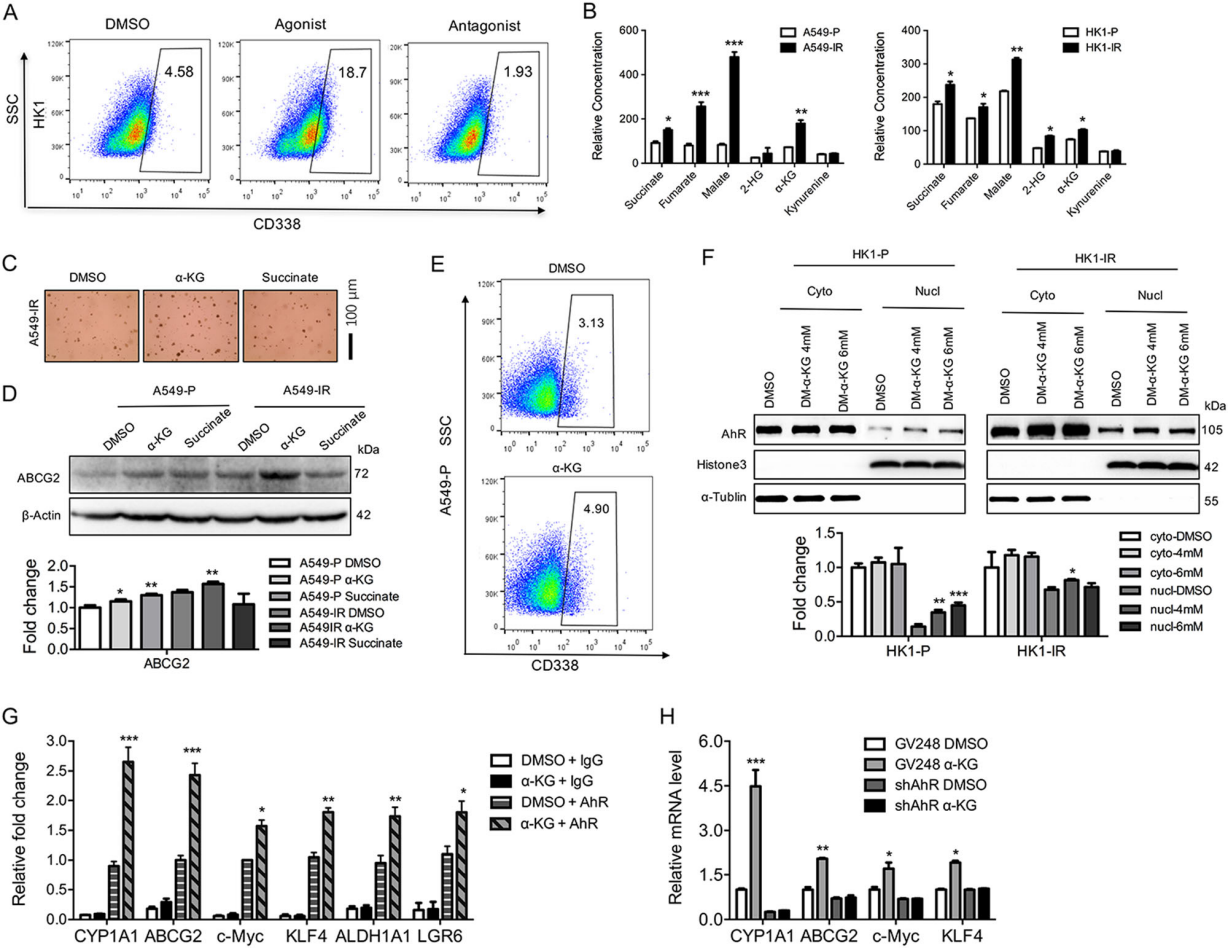
Activation of AhR and α-ketoglutarate are linked to stem-like properties in radioresistant sublines. **a** Representative images of flow cytometry analysis for the detection of CD338-positive cells in HK1 after treatment with AhR agonist and antagonist (*n* = 3). **b** GC-MS measured the indicated TCA metabolites in A549-IR (left) and HK1-IR (right) cells and P lines (*n* = 6). **c** Soft-agar assays were analyzed in A549-IR after the addition of α-KG and succinate (*n* = 3). **d** Western blot analysis was detected in A549-P and A549-IR sublines after the addition of α-KG and succinate (*n* = 3). The mean values of western blot quantification are shown at the bottom. **e** Representative images of flow cytometry analysis for the detection of CD338-positive cells in A549-P after the addition of α-KG (*n* = 3). **f** Nuclear and cytoplasmic fractions of HK1-P and HK1-IR lines showed elevated AhR in the nucleus after the treatment with α-KG (*n* = 3). The mean values of western blot quantification are shown at the bottom. **g** ChIP analysis in HK1-P sublines was performed to detect AhR binding to stemness-related genes as indicated after treatment with α-KG (*n* = 4). **h** RT–PCR analysis in A549-IR sublines in the depletion of AhR was used to detect the stemness-related genes as indicated after the addition of α-KG (*n* = 3); *p < 0.05, **p < 0.01, ***p < 0.001

Given the link between reduced AhR expression and decreased stem-like properties, we assessed whether increased AhR activation might inversely modulate the process. When A549-IR and H358-IR cells were treated with an AhR agonist and antagonist, we found that CYP1A1 mRNA levels were modulated after treatment with the AhR agonist in A549-IR and H358-IR cells and the P cells (Supplementary Figure S6A-B), treatment with the AhR agonist also elevated ABCG2 mRNA (Supplementary Figure S6A-B). Furthermore, treatment of A549-IR cells with an AhR agonist elevated the percent of CD338+ cells, whereas the AhR antagonist decreased the number of CD338+ cells (Fig. 5a). Treatment with the AhR agonist elevated the ABCG2 protein levels in HK1 and H358 P cells as well as H358-IR cells (Supplementary Figure S6C-D), moreover, the AhR agonist elevated the protein levels of ABCG2, c-Myc, and KLF4 in A549 P cells (Supplementary Figure S6E). The soft-agar assay showed that AhR agonist, not AhR antagonist, treatment increased colony formation in A549-IR cells (Supplementary Figure S6F). Moreover, stemness-related genes including ABCG2, c-Myc, and KLF4 were not induced by AhR agonist after depletion of AhR in both HK1-IR (Supplementary Figure S6G) and A549-IR cells (Supplementary Figure S6H) at mRNA levels, indicating that the induction of stemness-related genes is dependent on the activation of AhR signaling pathway.

The AhR pathway can cross talk with other major signaling pathways that might be modulated by oncometabolites that are critical in cancer progression^21–23^. We found elevated oncometabolite levels including 2-hydroxyglutarate (2-HG), α-ketoglutarate (α-KG), citrate, fumarate, malate, and succinate in A549-IR and HK1-IR cell sublines compared to P lines (Fig. 5b, c). Furthermore, after addition of α-KG, we observed that the colony numbers increased in A549-IR cells, whereas addition of succinate did not change the colony number in the soft-agar assay (Fig. 5d). Interestingly, the addition of α-KG, not succinate, triggered the activation of AhR through detection of CYP1A1 (Supplementary Figure S7B). Furthermore, addition of α-KG, but not succinate, induced an increase of ABCG2 mRNA and protein levels in A549-IR and P lines (Fig. 5e and Supplementary Figure S7C), and flow cytometry showed that α-KG increased the percent of CD338+ cells from 3.13 to 4.9 (Fig. 5f), indicating that α-KG might induce cancer stem-like properties through triggering AhR signaling pathway. As AhR is localized in the nucleus as an active form, we found that the nuclear fraction of AhR was indeed increased in A549-P and HK1-P cells with the addition of α-KG (Fig. 5g). Using ChIP analysis, we observed increases in AhR occupancy at the promoter region of several stemness-related genes including ABCG2, c-Myc, KLF4, ALDH1A1, and LGR6 after the addition of α-KG (Fig. 5h). Interestingly, addition of α-KG increased the mRNA expression of CYP1A1, ABCG2, c-Myc, and KLF4 in A549-P cells, this increase was abolished after depletion of AhR using shRNA knockdown (Fig. 5i), indicating a functional link between α-KG and AhR in a dependent manner. In summary, activation of the AhR is linked with stem-like property.

### Nuclear IKKα involves in the regulation of AhR in stemness-related genes

**Fig. 6:**
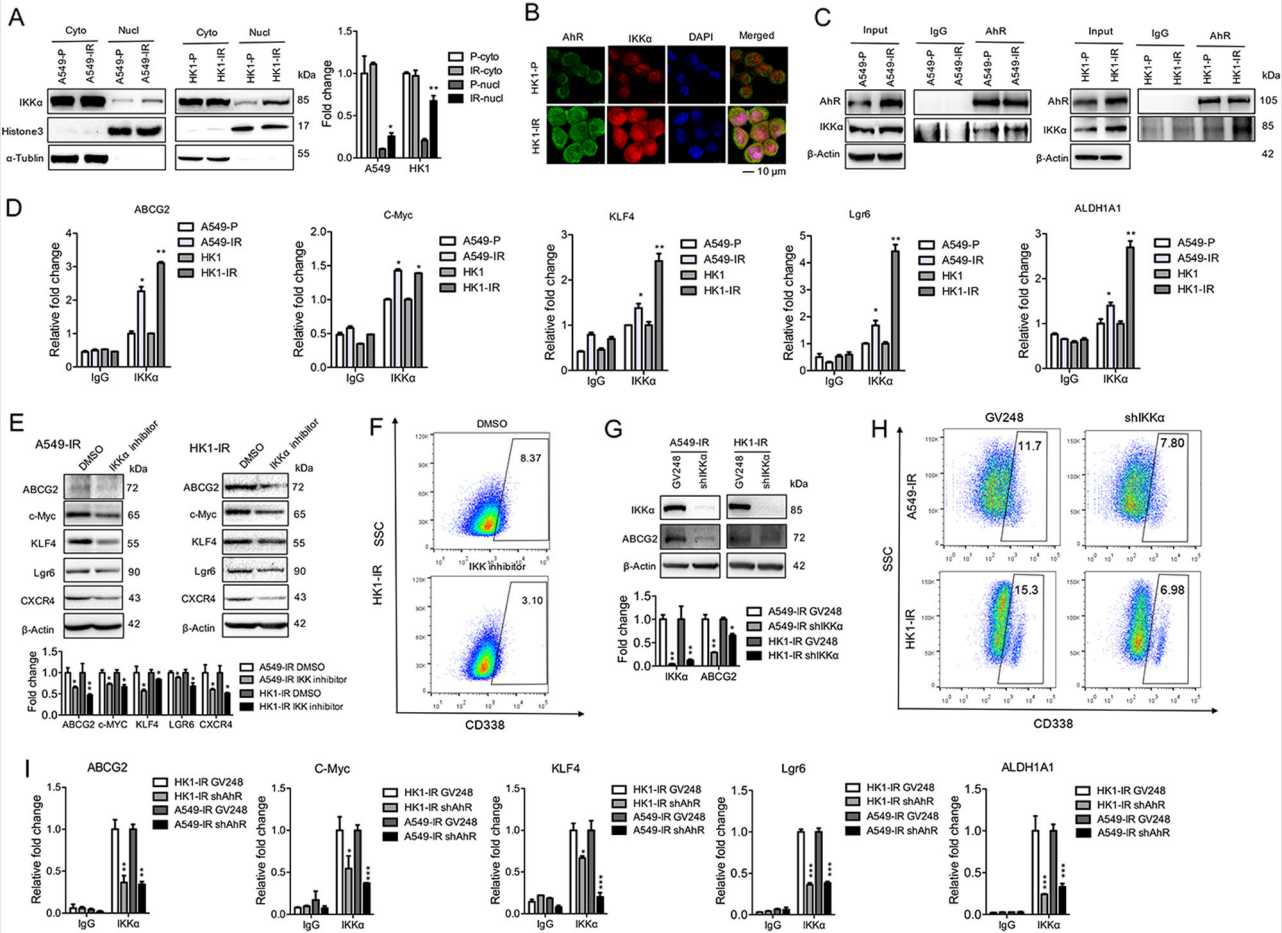
Nuclear IKKα involves in the regulation of AhR in stemness-related genes. **a** Western blot analysis was used to detect AhR and IKKα in the cytoplasm and nuclear protein from A549-IR and HK1-IR cells and their P lines (*n* = 3). The mean values of western blot quantification are shown on the right. **b** AhR co-localizes with IKKα in HK1-IR and HK1-P cells by shown by direct immunofluorescence analysis (*n* = 3). **c** Co-IP was used to detect the intact complex of AhR and IKKα elevated in A549-IR (left) and HK1-IR (right) sublines compared to P lines (*n* = 3). **d** ChIP analysis in A549-IR and HK1-IR sublines was performed to detect IKKα binding to stemness-related genes as indicated (*n* = 4–5). **e** The protein levels of stemness genes as indicated were detected by Western analysis after treatment with IKKα inhibitor (10 μM) for 72 h in A549-IR (left) and HK1-IR (right) sublines. The mean values of western blot quantification are shown at the bottom. **f** Representative images of flow cytometry analysis for detection of CD338-positive cells in HK1-IR sublines after the treatment with IKKα inhibitor (10 μM) for 72 h (*n* = 3). **g** The protein levels of IKKα and ABCG2 were detected by Western analysis in the depletion of IKKα in A549-IR (left) and HK1-IR (right) sublines (*n* = 3). The mean values of western blot quantification are shown at the bottom. **h** Representative images of flow cytometry analysis for the detection of CD338-positive cells in A549-IR (up) and HK1-IR (bottom) sublines in the depletion of IKKα (*n* = 3). **i** ChIP analysis in A549-IR and HK1-IR sublines in the depletion of AhR was performed to detect IKKα binding to stemness-related genes as indicated (*n* = 4); *p *<* 0.05, **p *<* 0.01, ***p *<* 0.001

We have recently shown that IKKα can localize to the nucleus and directly bind to the target gene’s promoters^13,24,25^. Since we observed that the nuclear levels of IKKα increased in A549-IR and HK1-IR cells compared to P cells (Fig. 6a), we tested whether IKKα and AhR may form an intact complex. Using immunofluorescence assay, we found that IKKα colocalized with AhR in the nucleus (Fig. 6b); we further found evidence of IKKα and AhR interaction in IR sublines and P lines using co-immunoprecipitation assay, whereas the intact complex of IKKα and AhR formed in A549-IR and HK1-IR cells as compared to P cells (Fig. 6c). On the basis of higher nuclear localization of IKKα in radioresistant sublines, ChIP analysis demonstrated an increased enrichment of IKKα at the promoter of stemness-related genes including ABCG2, c-Myc, KLF4, Lgr6, and ALDH1A1 in A549-IR and HK1-IR cell sublines compared to P lines (Fig. 6d), indicating that IKKα directly involves in the regulation of stemness genes. Furthermore, in order to investigate whether the binding of IKKα at the promoter of stemness-related genes is dependent of AhR, we performed ChIP analysis and found that the enrichment of IKKα decreased at the promoter of stemness-related genes in HK1-IR and A549-IR cells after depletion of AhR (Fig. 6e), indicating that AhR is essential for IKKα recruitment.

To further confirm the critical role of IKKα in the regulation of stemness genes, we first treated A549-IR and HK1-IR cells with IKKα inhibitor, IKK inhibitor XII, a selective inhibitor of IKKα activity. Figure 6f showed that the inhibitor of IKKα reduced the protein levels of stemnes genes including ABCG2, c-Myc, KLF4, Lgr6, and CXCR4. FACS assay showed that the IKKα inhibitor lead to a decrease of CD338+ expression in HK1-IR cells compared to P lines (Fig. 6g). Moreover, depletion of IKKα also decreased ABCG2 expression in A549-IR and HK1-IR cells (Fig. 6h); also, knockdown of IKKα decreased the percent of CD338 positive in A549-IR and HK1-IR cells (Fig. 6i). Taken together, these data suggest a critical role of IKKα in the regulation of stemness-related genes in radioresistant cells through an interaction with AhR.

**Fig. 7:**
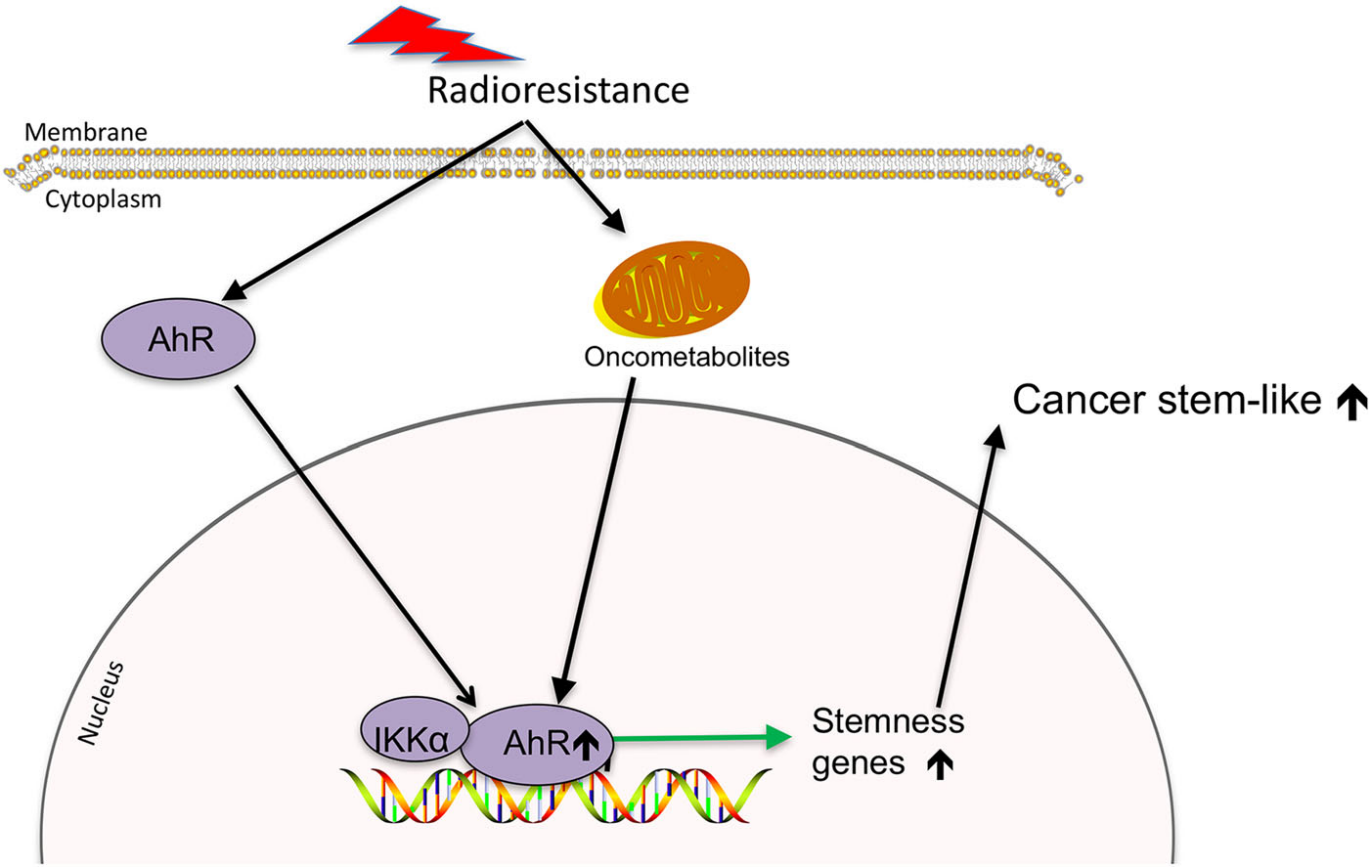
The schematic model of AhR in tumorigenic, stem-like, metastasis properties in the occurrence of radioresistance. AhR induces stem-like properties by directly targeting the promoters of stemness-related genes; the intact complex of IKKα and AhR also directly activates the stemness-related genes. Increased generation of oncometabolites in radioresistant sublines, in turn, induces stem-like signature gene expression in an AhR-dependent manner

Based on our findings, we propose a model (Fig. 7). In this model, AhR acts as a core component of radioresistance involved in tumorigenic, stem-like and metastatic properties.

## Discussion

Approximately 50% of all cancer patients receive radiotherapy, and it is worth mentioning that about 20% of those patients develop local recurrence which has been linked to the acquisition of radioresistance^14^. Several tumor-promoting genes such as CXCR4, LSH, MMP9, and hypoxia-inducible factor (HIF)-1α are thought to contribute to radioresistance, and their elevated expression is consistent with their proposed role in cancer progression, metastasis, and radiotherapy^24,26–30^.

AhR, a widely expressed nuclear receptor that senses environmental stimuli and modulates target gene expression, plays a critical role in breast CSCs etc^31,32^. AhR influences the major stages of tumorigenesis and chemoresistance^33–35^, and studies of aggressive tumors and tumor cell lines have shown increased levels of AhR protein and constitutive nuclear localization in cancer tissue, whereas in normal tissues AhR is mainly inactive and resides in the cytoplasm^6,7^. Here we found elevated AhR level in the nucleus linked with radioresistance in LC and NPC cells, indicating that activation of AhR contributes to cancer progression, stem-like properties, and radioresistance. Nuclear IKKα transcriptionally upregulates several miRNAs, contributing to chemoresistance^36^. Recently, mixed lineage kinase 4 binds and phosphorylates IKKα, leading to activation of NF-κB signaling in glioma stem cells^37^. Here we show that nuclear IKKα targets the promoters of stem-like genes as a partner of AhR, contributing to radioresistance, indicating a critical role of nuclear IKKα in cancer progression and radioresistance. Moreover, we found that the protein level of AhR, not mRNA level, increased in radioresistant sublines.

There is an opposed effect of IKKα in several types of cancer in the process of carcinogenesis. For example, inhibition of IKKα prolongs survival and suppresses the occurrence of metastatic diseases in models of mammary, prostate, and colorectal cancers^38–42^, whereas, IKKα controls metastasis in breast and prostate cancers and is required for mammary tumorigenesis through expansion of tumor-initiating cells^39–41^. In contrast, IKKα acts as a tumor suppressor in models of skin or lung (Squamous cell carcinoma) SCC, while loss of IKKα enhances susceptibility to carcinogen-induced SCC in the skin and leads to the development of spontaneous lung SCC^43,44^. Recently, we show that IKKα is diversely expressed in keratinizing and non-keratinizing carcinomas in the same type of cancer^13^. We also provide evidence that IKKα is absent in SCC of the skin while it is expressed in (Basal-cell carcinoma) BCC, indicating the opposed roles of IKKα in skin cancer between SCCs and BCCs, which originate from both keratinocyte tumors^25^. Moreover, SCC development is generally associated with cell dedifferentiation that IKKα is reversely involved in the process. Here we show the critical role of IKKα in the regulation of stemness-related genes in radioresistant cells through an interaction with AhR.

Epithelial–mesenchymal transition (EMT) is a key factor of cancer progression, metastasis, and self-renewal of CSCs^45–47^. TGF-β tumor suppression functions through an EMT-mediated disruption of a lineage-specific transcriptional network^48^. Interestingly, EMT is not required for metastasis but contributes to chemoresistance^49,50^. However, we did not find the classical features of EMT and (mesenchymal-epithelial transition) MET in the stem-like radioresistant cells, but a hybrid EMT/MET state (data not shown). This EMT/MET hybrid state is thought to be bidirectional displaying a gradient of partial states toward either extreme EMT or extreme MET, and further supports the importance for metastasis and metastasis-initiating cells as well as circulating tumor cells in the epithelial and mesenchymal composition^51–54^, and it also supports that an intermediate EMT state may make cells more prone to exhibiting stem-like properties^54^. Our findings indicate that stemness seems to be better manifested at intermediate epitheloid states, consistent with those observed in cultured mammospheres. In particular, tumor-associated fibroblasts may be responsible for inducing EMT by secreting TGF-β that can stimulate proliferation and lead to expansion of the preexisting CSC pool^55,56^, even though we did not find that TGF-β changed in radioresistant cells.

The AhR pathway can cross talk with other major signaling pathways that might be modulated by oncometabolites that are critical in cancer progression^21–23^. Diverse metabolites serve as cofactors or substrates for enzymes that are involved in the deposition or exchange of epigenetic marks, driving a metabolite-driven pathway of gene regulation as well as distinct cancer tissue types^24,57–59^. Also, organ-specific differences were observed in the metabolite levels of the TCA cycle and other intermediates^58,60^. 2-HG is a competitive inhibitor of α-KG-dependent dioxygenases in gliomas and hematological malignancies that carry mutations of isocitrate dehydrogenase genes (IDH1 and IDH2)^61,62^. However, we did not find mutations of IDH1 and IDH2 genes and accumulation of 2-HG in NPC and LC (data not shown). We found that the level of kynurenine, which is involved in tryptophan catabolism, remained the same in radioresistant sublines compared to P sublines, indicating that kynurenine did not further activate AhR signaling pathway, but increased the generation of α-KG oncometabolite in radioresistant sublines, and in turn induced stem-like signature gene expression by AhR in a dependent manner. Here, we report that α-KG can activate the AhR signaling pathway leading to elevated stemness gene expression to influence the pluripotency state, and we provide evidence of a direct link between cellular metabolism, AhR signaling, and radioresistance associated with stem-like property.

Taken together, we elucidated the crucial role of AhR in the maintenance of radioresistance and stem-like property involving in direct activation of self-renewal via stemness genes, leading to metabolic reprogramming. Our data showed that stemness reprogramming controlled by metabolism can modulate radioresistance and tumor progression.

## Material and methods

### Cell culture, antibodies, plasmids, siRNAs, and chemicals

Lung adenocarcinoma cell lines A549-P/H358-P and its irradiation-resistant cell lines A549-IR/H358-IR were provided by Xinming Deng (Emory University School of Medicine and Winship Cancer Institute of Emory University, Atlanta, USA). A549-P/IR cell lines were maintained in DMEM/F12 and RPMI 1640 medium, respectively, which were supplemented with 10% fetal bovine serum (FBS). NPC cell line HK1 was used to establish ionizing radiation resistant cell line (HK1-IR), as described^63^. Briefly, HK1 cells were serially irradiated with 4 Gy of X-rays to a final dose of 80 Gy using XRAD320 (Precision X-ray, Inc., North Branford, CT). HK1-P/IR cell lines were maintained in RPMI 1640 medium. All cell lines tested negative for mycoplasma contamination and were maintained at 37 °C with 5% CO_2_. All cell lines were authenticated by short tandem repeat profiling and were passaged less than ten times after initial revival from frozen stocks.

Primary antibodies as indicated for Western analysis were purchased from Cell Signaling (Danvers, MA). Primary antibodies for AhR and β-actin were purchased from Sigma-Aldrich (St. Louis, MO). GV248-shAhR lentivirus plasmids were purchased from Genechem (www.genechem.com.cn). RNAi sequences used in these studies are provided (shAhR-1: CACAACAATATAATGTCTT; shAhR-2: TTCTTTGATGTTGCATTAA; shAhR-3: AATGATTAAGACTGGAGAA; shAhR-4: ATAATAACTCCTCAGACAT). The chemicals, citrate, α-KG, 2-HG, fumarate, malate, succinate, oxalacetic acid, 2-isopropylmalic acid, methanol, N,O-bis(trimethylsilyl) trifluoroacetamide, and trimethylcholorosilane and its dimethyl products were purchased from Sigma-Aldrich (St. Louis, MO). The chemicals AhR agonist V, VAF347 (Cat# 182690), and AhR antagonist (Cat# 182705) were purchased from Calbiochem.

### Western blot analysis and co-immunoprecipitation (Co-IP) assay

Details of the western blot analysis and Co-IP assay were described previously^24,29^. A detailed procedure is presented in the Supplementary Material and Methods section. The following list of antibodies was used for western blot detection: AhR and IKKα antibodies.

### Quantitative real-time PCR

Cells were harvested with Trizol (Invitrogen). cDNAs were synthesized with SuperScript III (Invitrogen) according to the manufacturer’s protocol. Real-time PCR analysis was performed using the Applied Biosystems 7500 Real-Time PCR System, according to the manufacturer’s instructions. The reactions were performed in triplicates for three independent experiments: the results were normalized to β-actin. For qPCR, an SYBR Green-based method was used and the relative quantitation of gene expression was determined using the comparative CT (^ΔΔ^CT) method and normalized to the β-actin gene.

The PCR primer sequences used are given in the Supplementary Table S1. The mean ± SD of three independent experiments is shown.

### Cell proliferation assay, migration and invasion assay, and plate-colony formation assay

Details of the cell proliferation assay were described previously^29^. For plate-colony formation assay, cells (2 × 10^3^/ml/well) were seeded into 6-well plates and cultured in RPMI-1640 medium supplemented with 10% FBS. Colonies were fixed with methanol and stained with viola crystalline, then scored using a microscope and Image J software (1.47V, NIH, USA).

The migration assay was described previously^24^. Cells (5 × 10^5^) were seeded onto the upper chamber in 200 μl of serum-free medium; the lower compartment was filled with 0.6 ml of DMEM media supplemented with 10% of FBS. After 24 h incubation, migrated cells on the lower surface of the filter were fixed and stained using propidium iodide. Cells on the upper side were removed using a rubber scraper. Fluorescent images were obtained. The reported data represent the counts of migrated cells. Experiments were performed in triplicates.

For the plate-colony formation assay, cells (1 × 10^3^/ml/well) were seeded into 6-well plates and cultured in RPMI-1640 medium supplemented with 10% FBS. Colonies were fixed with methanol, stained with viola crystallina and scored using a microscope and ImageJ software (1.47V, NIH, USA). Surviving colonies were counted and the surviving fraction (SF) was calculated using the formula SF = treatment colony numbers/control colony numbers.

### Soft-agar colony forming assay

To assess anchorage-independent growth, soft-agar colony forming assay was performed using 8000 cells per well. A layer of agar containing 3.0 ml of 0.6% soft agar (BD Biosciences, USA) in (basement membrane extract) BME was poured into wells of a 6-well cell culture dish and allowed to set at room temperature for 30 min. A second layer containing 1 ml 0.35% soft agar in BME containing cells (800 cells/ml) was placed on the top of the first layer and allowed to set at room temperature for 30 min. Cells were incubated in an incubator at 37 °C for 14 days, and the number of colonies were counted, and the images were captured using an Olympus microscope.

### Oncosphere formation assay

Cells were seeded on ultra-low attachment culture dishes (Corning, Corning, NY) in serum-free DMEM-F12 medium containing 50 μg/ml insulin (Sigma-Aldrich St. Louis, MO), 0.4% Albumin Bovine Fraction V (Sigma-Aldrich St. Louis, MO), N-2 Plus Media Supplement (Life Technologies, Grand Island, NY), B-27 Supplement (Life Technologies, Grand Island, NY), 20 μg/ml EGF (PeproTech Rocky Hill, NJ), and 10 μg/ml basic FGF (PeproTech, Rocky Hill, NJ) to support the growth of undifferentiated oncospheres. Cells were incubated in a CO_2_ incubator for 1–2 weeks, and the numbers of oncosphere cells were counted under a microscope.

### Immunofluorescence assay and Operetta® High Content Screening and High Content Analysis

Details of the immunofluorescence assays were described previously^24,29^, cells were cultured and fixed in 4% paraformaldehyde for 30 min. To identify the potential presence of AhR and IKKα, cells were incubated with an anti-AhR antibody (Sigma) and an anti-IKKα antibody (Active motif) and then with fluorescein isothiocyanate (FITC)-conjugated anti-IgG (Santa Cruz) and Cy3-conjugated anti-IgG (Sigma). To visualize the nuclei, the cells were stained with Hoechst (1:1000). Fluorescent images were observed and analyzed with a laser scanning confocal microscope (Bio-Rad MRC-1024ES).

For Operetta® High Content Screening and High Content Analysis, cells were grown on the 96-well plate. After incubation overnight, the cells were washed with phosphate-buffered saline (PBS) and fixed with methanol for 10 min at 37 °C. The cells were incubated in PBS supplemented with 1% BSA for 1 h at room temperature. The cells were incubated overnight with E-cadherin mouse monoclonal antibody (1:100 dilution, abcam 1416) or Vimentin rabbit monoclonal antibody (1:200 dilution, CST 5741s) at 4 °C. The cells were washed three times in PBS and then stained with Alexa Fluor 594 Goat Anti-Mouse IgG or Alexa Fluor 594 Goat Anti-Rabbit IgG (1:4000 dilution, Invitrogen) for 1 h at room temperature. After incubation with DAPI to stain the nuclei, the cells were imaged on the Operetta® High Content Imaging System and analyzed using Harmony® High Content Image Analysis Software (USA).

### ALDEFLUOR assay, flow cytometry, and cell sorting

Cells were stained directly using different antibodies (including CD338-PerCP-Cy™5.5, CD326-BV510) according to the manufacturer’s instructions. Labeled cells were detected using a FACSCalibur (BD Immunocytometry Systems, CA, USA) and analyzed with Flowjo software. IgG isotype controls corresponding to each directly conjugated fluorophore were utilized to identify, quantify, and positively select desired cell populations. Debris and cell clusters were excluded during side-scatter and forward-scatter analyses. For cell sorting, cells were stained and followed by sorting with FACS Aria III (BD Immunocytometry Systems, CA, USA). The separated populations were used for RNA and animal experiments.

The ALDEFLUOR kit (StemCell Technologies Inc.) was used as described previously^64^ and with the guidance of Dr. Suling Liu (Fudan University, Shanghai, China).

### Chromatin immunoprecipitation (ChIP) assays

ChIP assays were essentially performed as previously described^24,29^ with modifications: 5 × 10^6^ cells were fixed with formaldehyde (1% final volume concentration, Sigma), 10 min at room temperature. Fixation was stopped by the addition of 1/10 volume 1.25 M glycine and incubated for 5 min at room temperature. The sonication step was performed in a Qsonica sonicator (5 min, 20 s on, 20 s off), and 200 μg of protein–chromatin complex was used for each immunoprecipitation. Antibody–protein complex was captured with preblocked dynabeads protein G (Invitrogen). ChIP DNA was analyzed by qPCR with SYBR Green (Biorad) in ABI-7500 (Applied Biosystems) using the primers specified in Supplemental Table S2. The antibodies used are as indicated in Supplemental Table S3.

### Nude mice and study approval

A xenograft tumor formation was essentially performed as previously described^26^, whereas the Hunan SJA laboratory Animal Co. Ltd. (http://www.hnsja.com) provided the mice. SCID mice were injected with indicated cells (1 × 10^6^ cells/mouse or as indicated) via mammary fat pad or tail vein (6 mice/group or as indicated). Mice with A549-IR cells or indicated cells were imaged from dorsal and ventral views once per week. All mice (6 of 6) injected with cells via the tail developed the expected lung metastatic lesions within 8 weeks. Visible lung metastatic nodules were examined macroscopically or detected in paraffin-embedded sections stained with H&E. Data were analyzed using Student’s *t*-test; a *p* value < 0.05 was considered significant.

All procedures for animal study were approved by the Institutional Animal Care and Use Committee of the Central South University of Xiangya School of Medicine and conform to the legal mandates and federal guidelines for the care and maintenance of laboratory animals.

NCI-Frederick is accredited by AAALAC International and follows the Public Health Service Policy for the Care and Use of Laboratory Animals. Animal care was provided in accordance with the procedures outlined in the “Guide for Care and Use of Laboratory Animals (National Research Council; 1996; National Academy Press; Washington, DC).

### Immunohistochemistry (IHC) analysis and in situ hybridization of tumor biopsies

NPC biopsies, validated by pathologist Dr. Desheng Xiao (Xiangya Hospital), were obtained from the Department of Pathology of Xiangya Hospital. The NPC tissue array was purchased from Pantomics (Richmond, CA, USA). IHC analysis of paraffin sections from NPC patient or xenograft samples was performed as described previously^26,65^. In situ hybridization was performed using the EBV-encoded RNA (EBER) HRP conjugated probe and DAB as substrate from the ISH kit (Life technologies), according to the manufacturer’s instructions.

### Statistics

The experiments were repeated at least three times except the nude mice experiments. Results are expressed as mean ± SD or SEM as indicated. All statistical analyses were performed using Prism 6.0 GraphPad Software. Significant differences between two groups were compared using the Student’s *t*-test, and comparisons among more than two groups were performed using analysis of variance (ANOVA). The correlation analysis was conducted using Pearson’s correlation coefficient. A *p* value less than 0.05 was considered statistically significant.

Additional information can be found in the Supplementary Materials and Methods section.

## Electronic supplementary material

Supplementary Figures, Tables and Material and Methods
