# Supplementary material for: Activation of AhR with nuclear IKKα regulates cancer stem-like properties in the occurrence of radioresistance
Source: Cell Death Dis. 2018 Apr 30;9(5):490. doi: 10.1038/s41419-018-0542-9 (PMC5924755; doi:10.1038/s41419-018-0542-9)
Supplement: Supplementary file 1 — Supplementary Figures, Tables and Material and Methods [file 41419_2018_542_MOESM1_ESM.doc]

**Supplementary information**

**Activation of AhR with nuclear IKKα regulates cancer stem-like properties in the occurrence of radioresistance**

Bin Yan, Shuang Liu, Ying Shi, Na Liu, Ling Chen, Xiang Wang, Desheng Xiao, Xiaoli Liu, Chao Mao, Yiqun Jiang, Weiwei Lai, Xing Xin, Can-E Tang, Dixian Luo, Tan Tan, Jiantao Jia, Yating Liu, Rui Yang, Jun Huang, Hu Zhou, Yan Cheng, Ya Cao, Weishi Yu, Kathrin Muegge, Yongguang Tao

**Supplementary Figures and Figure Legends**


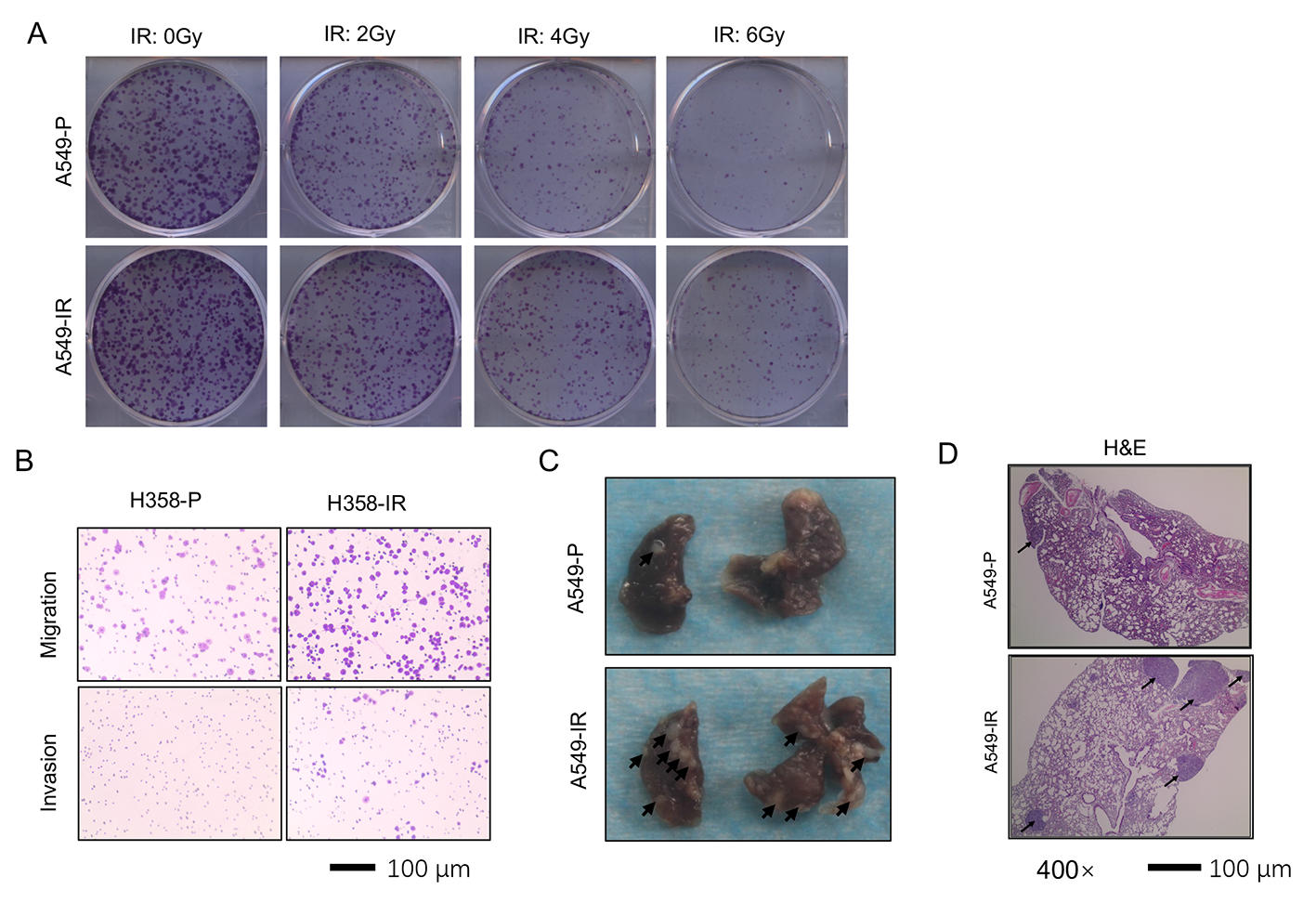


**Figure S1. Radiation resistant cells have more characteristics of cancer progression and metastasis, related to Figure 1.**

(A) Colony formation assay was analyzed in A549-P and A549-IR cells after the treatment of different dose of irradiation as indicated (*n*=3). (B) Migration and invasion assay were shown in H358-P and H359-IR cells (*n*=3). (C) Tumor formation in lung tissues from nude mice after the injection of A549-P and A549-IR cells into the tail vein of SCID mice for eight weeks. Arrow indicated tumors. (D) Tumors were shown by microscope in paraffin-embedded sections stained with H&E.


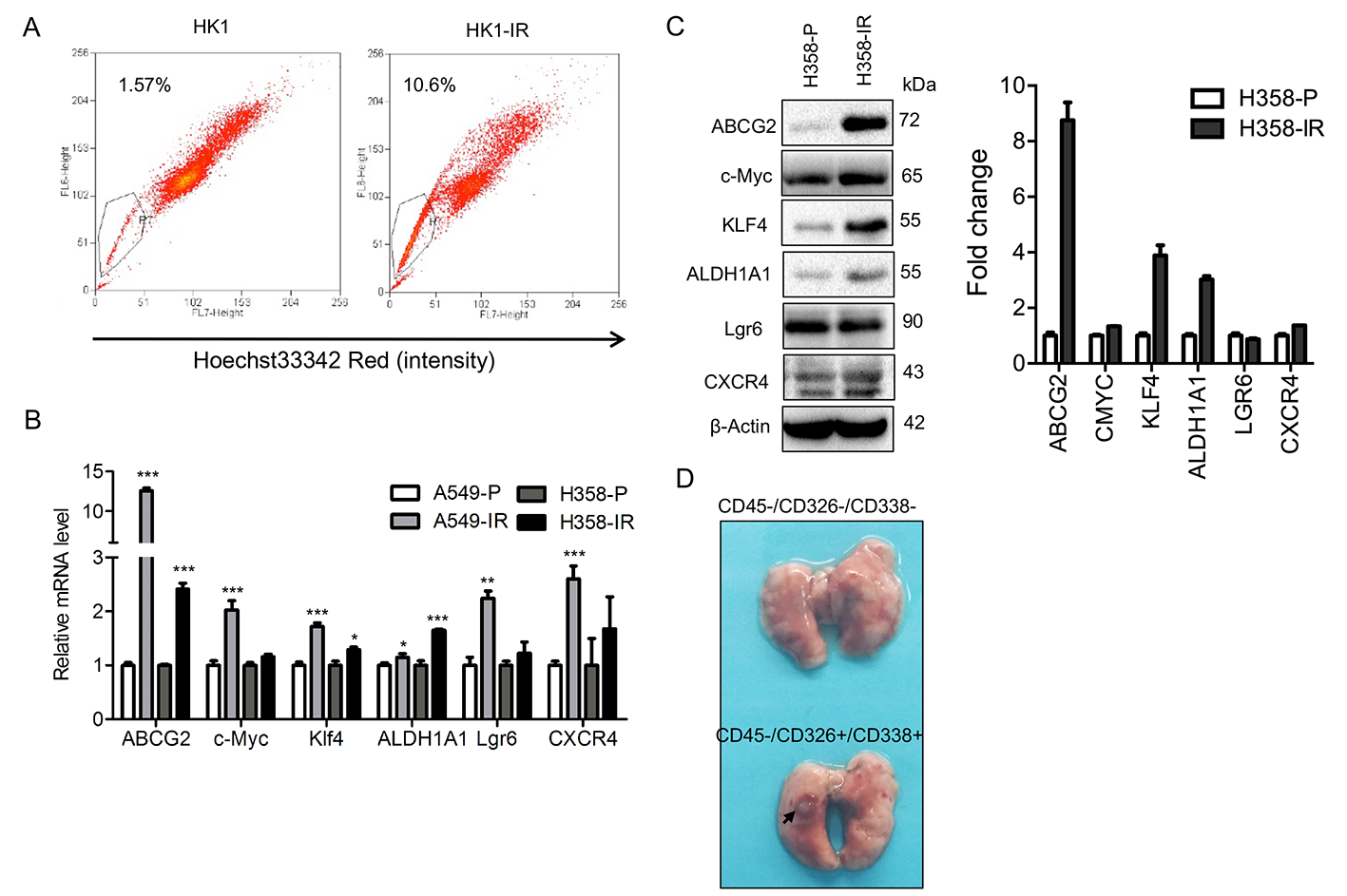


**Figure S2. Radiation resistant cells have more characteristics of stem-like properties, related to Figure 2.**

(A) Flow cytometry analysis showing side population cells from HK1-P and HK1-IR cells (*n*=3). (B) RT–PCR analysis for detection of the expression of stemness-related genes as indicated in A549-IR and H358-IR sublines compared to P lines (*n*=3). (C) Western Blot for detection of the expression of stemness-related genes as indicated in H358-P and H358-IR sublines (*n*=3). (D) Tumor formation in the lung after CD45-/CD326+/CD338+ cells derived from lung cancer patients were injected into the tail vein of SCID mice. Arrow indicated tumors. * *p* <0.05, ** *p* <0.01, *** *p* <0.001.


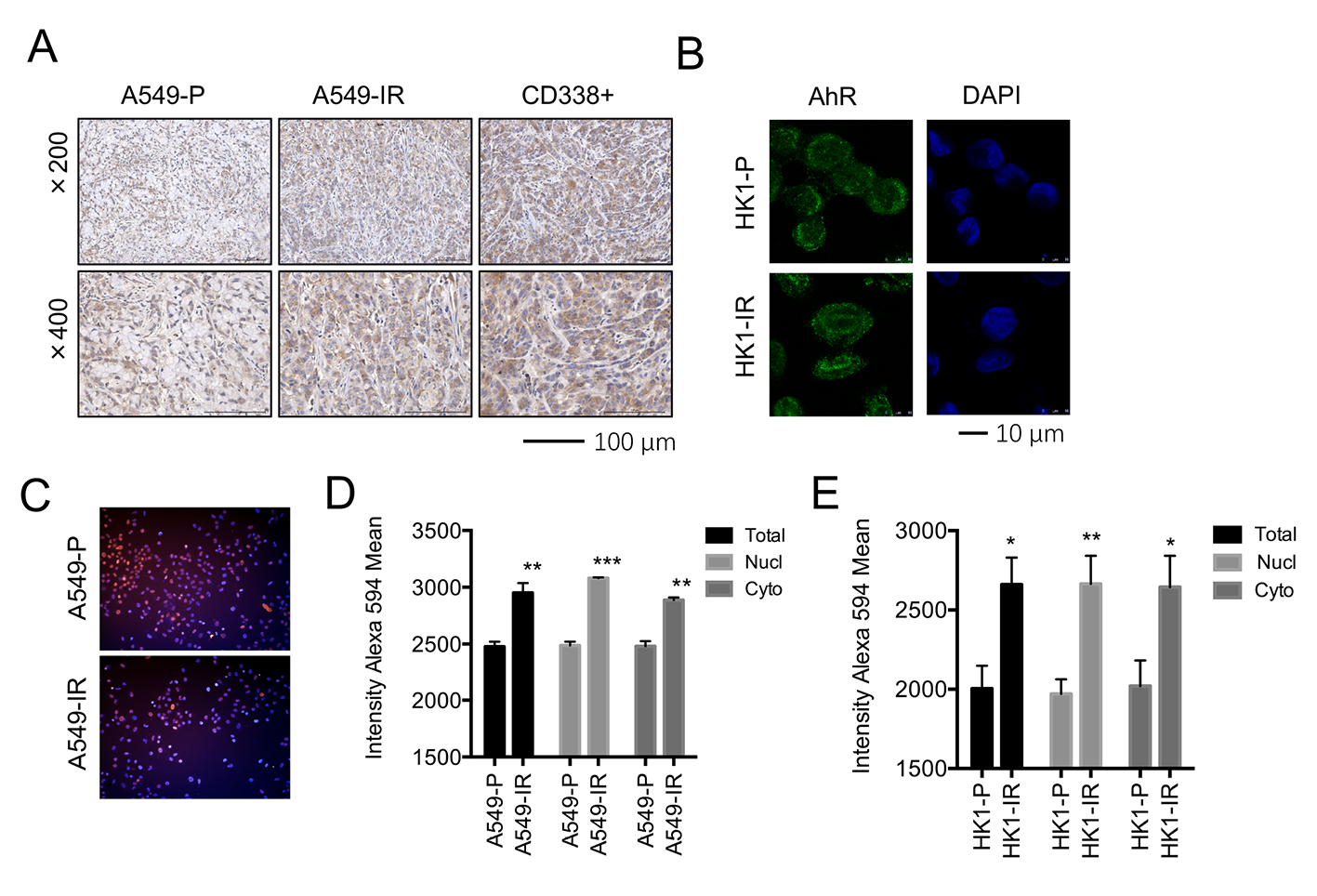


**Figure S3. AhR is elevated in radiation resistant cells, related to Figure 3.**

(A) Immunohistochemical analysis of AhR was used to determine AhR expression level as indicated in tumor samples generated in nude mice. (B) AhR localization in HK1-IR and HK1-P cells was determined by direct immunofluorescence analysis (*n*=3). (C) A representative experiment was shown for AhR in A549-P and A549-IR cells using high content screening and high content analysis. (D, E) Relative intensity of AhR in the cytoplasm and nucleus was shown in HK1-P, and HK1-IR cells (D), and A549-P, A549-IR cells (E) (*n*=5). * *p* <0.05, ** *p* <0.01, *** *p* <0.001.


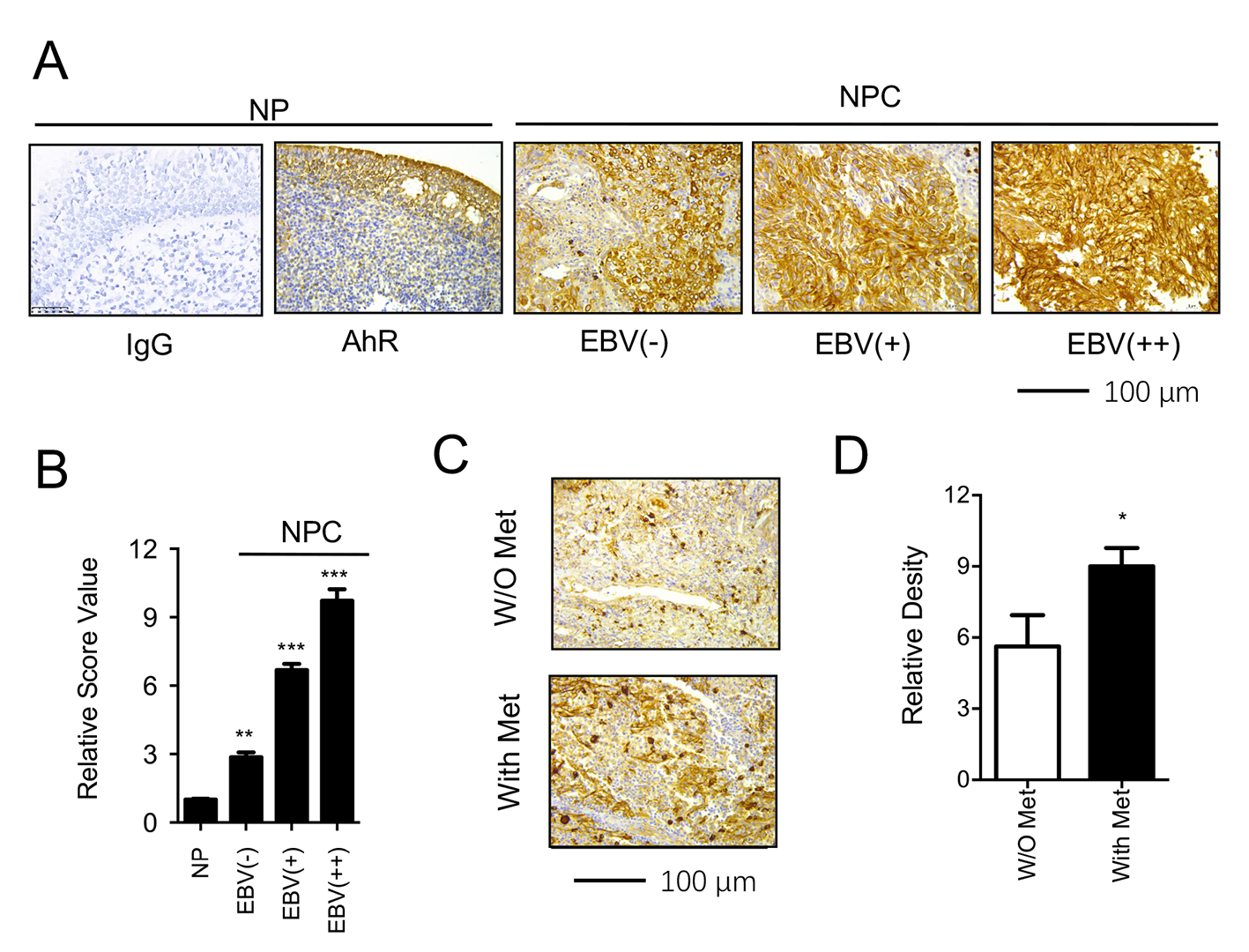


**Figure S4. AhR is elevated in NPC tissues, related to Figure 3.**

(A) Immunohistochemical analysis was used to determine the AhR protein level in an NPC tissue array from NPC patients. (B) AhR is expressed at low levels in inflamed nasopharyngeal tissues (NP, *n*=10), and at high levels in NPC tissues (NPC, *n*=87) with different levels of EBV-encoded RNA EBV (+, *n*=30) and EBV (++, *n*=28). (C, D) Immunohistochemical analysis of AhR was shown in NPC tissues of patients without metastasis (W/O Met) (*n*=16) and with metastasis (With Met) (*n*=14) (C) and anti-AhR staining intensity was quantified (D). * *p* <0.05, ** *p* <0.01, *** *p* <0.001.

**
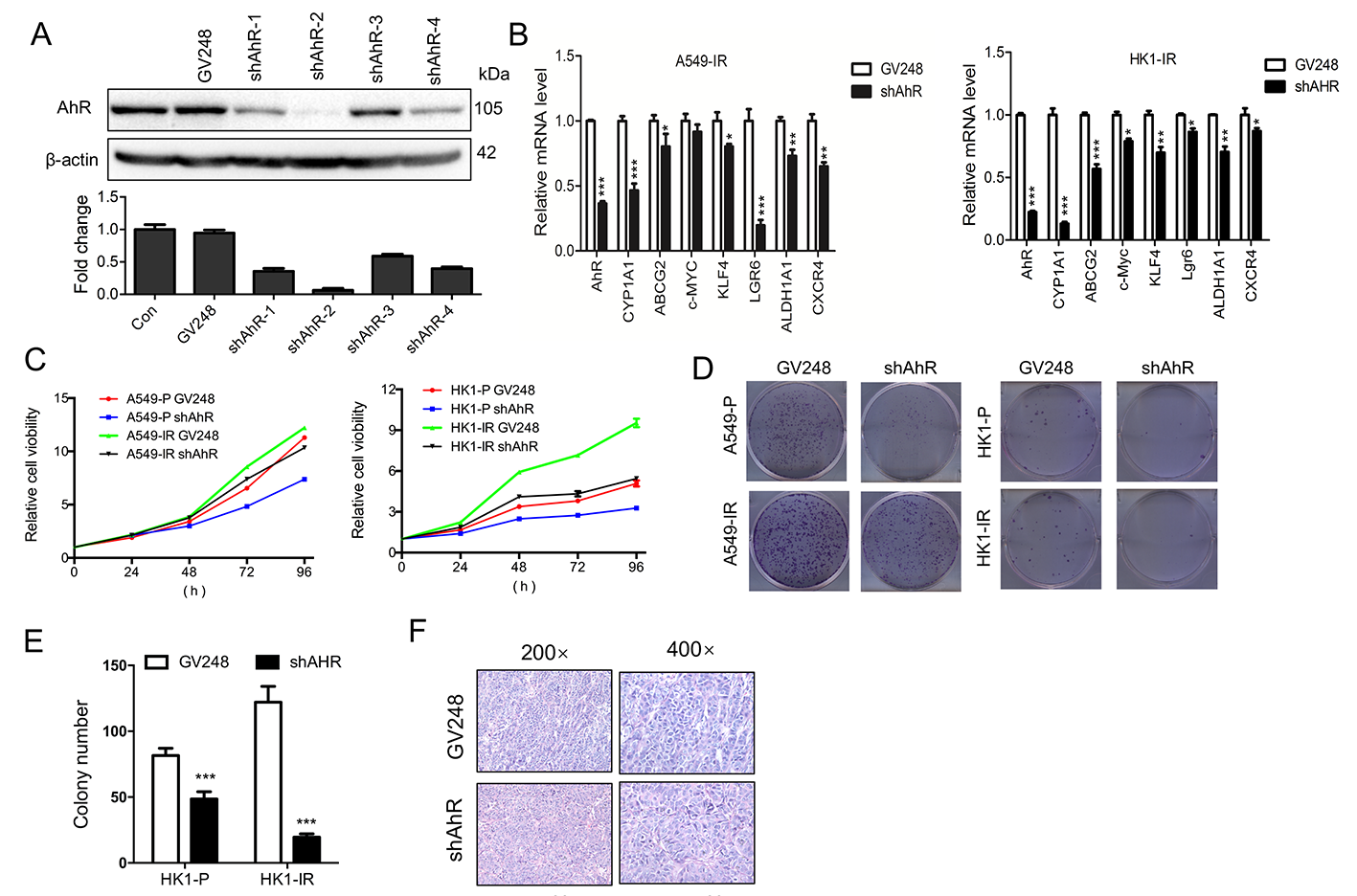
**

**Figure S5. Knockdown of AhR attenuates cancer progression, related to Figure 4.**

(A) AhR protein levels were analyzed by Western blot using different target sequence of shRNAs to AhR (*n*=3). (B) RT–PCR analysis from A549-IR (left) and HK1-IR (right) and sublines was used to determine the expression of stemness related genes as indicated after depletion of AhR (*n*=3). (C) The MTT assay was performed to assess cell viability in A549-IR (left) and HK1-IR (right) sublines after depletion of AhR (*n*=4). (D) Plate colony formation assay was measured in A549-P and A549-IR cells, HK1-P and HK1-IR cells in the depletion of AhR (*n*=3). (E) Relative colony number was shown in HK1-P and HK1-IR cells in the depletion of AhR using plate colony formation assay (*n*=3). (F) Animals were euthanized and assessed by microscope using paraffin-embedded sections stained with H&E. * *p* <0.05, ** *p* <0.01, *** *p* <0.001.

**
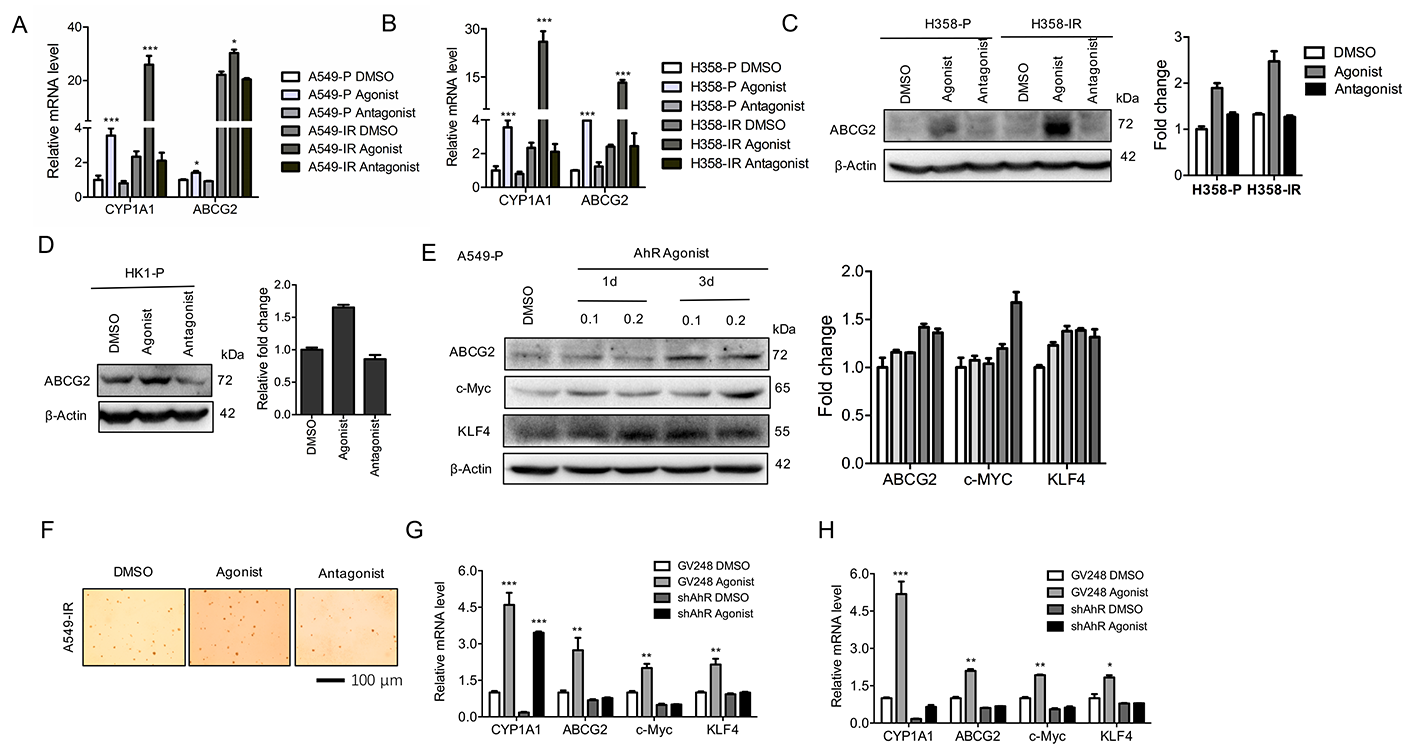
Figure S6. AhR signaling pathway is linked with stemness genes in the radioresistant sublines, related to Figure 4.**

(A, B) RT-PCR detected CYP1A1, ABCG2 in A549-IR (A) and H358-IR (B) cells after the treatment of agonist and antagonist (*n*=3). (C) Western blot analysis was used to detect ABCG2 in H358-P and A358-IR sublines after after the treatment of agonist and antagonist (*n*=3). (D) Western blot analysis was used to detect ABCG2 in HK1-P sublines after after the treatment of agonist and antagonist (*n*=3). (E) Western blot analysis was used to detect ABCG2, c-Myc and KLF4 in A549-P sublines after after the treatment of agonist (*n*=3). (F) Soft agar assay is shown as a representative experiment after the treatment of agonist and antagonist (*n*=3). (G, H) RT-PCR analysis was used to detected CYP1A1, ABCG2, c-Myc and KLF4 in A549-P (G), and HK1-P (H) in the depletion of AhR after the treatment of agonist and antagonist (*n*=3). * *p* <0.05, ** *p* <0.01, *** *p* <0.001.


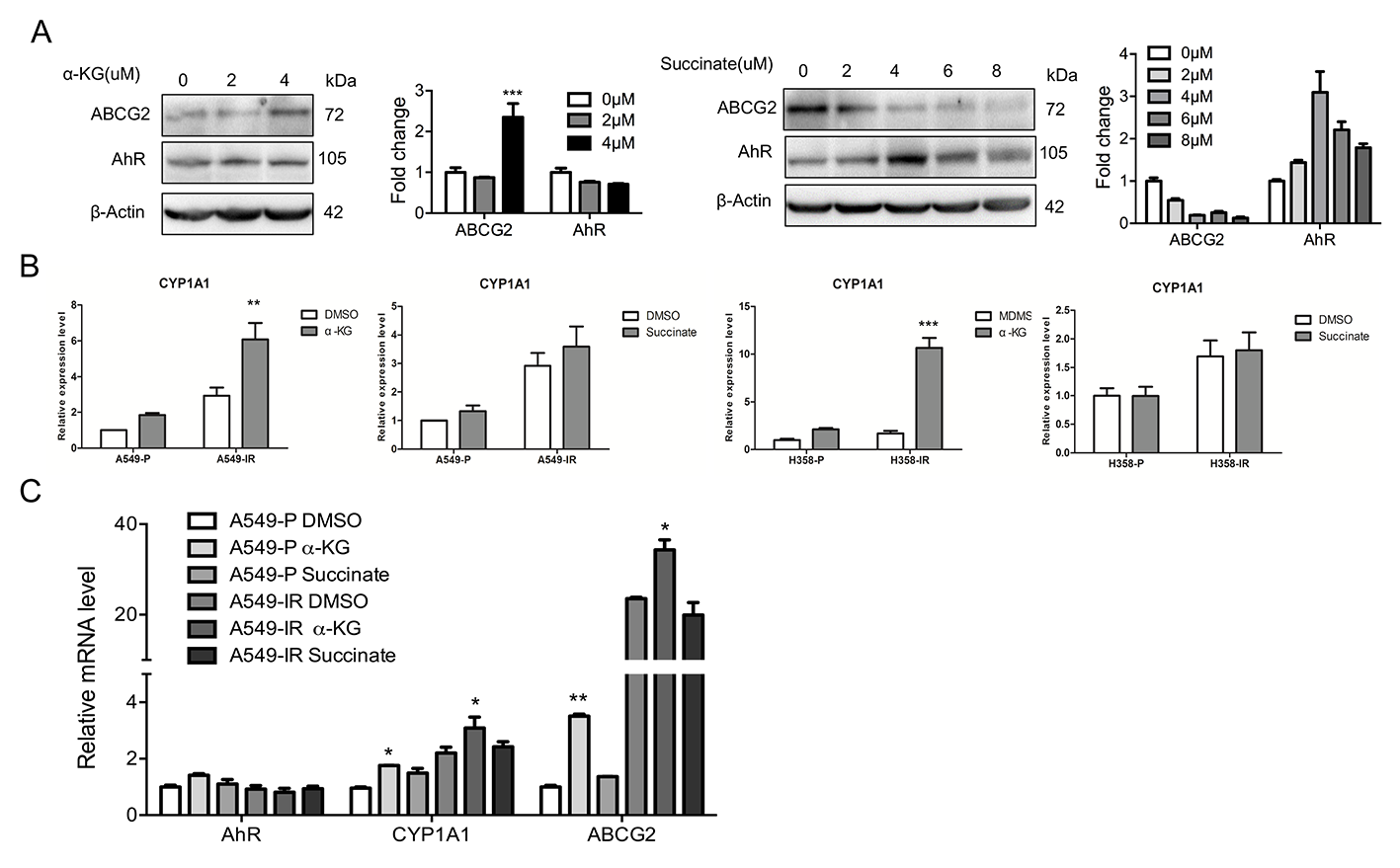


**Figure S7. Oncometabolite α-KG activates the AhR signaling and stemness genes, related to Figure 6.**

(A) Both ABCG2 and AhR protein levels were analyzed by Western blot after the treatment of α-KG and succinate (*n*=3). (B) RT-PCR detected CYP1A1 mRNA level in A49-IR and H358-IR after the treatment of agonist and antagonist (*n*=3). (C) RT-PCR detected the stemness genes as indicated in A549-P and A549-IR after the treatment of α-KG and succinate (*n*=3). * *p* <0.05, ** *p* <0.01, *** *p* <0.001.

**Table S1 RT-PCR primers.**

| Name | Full Name | Gene ID | Sequence | Product size(bp) |
| --- | --- | --- | --- | --- |
| AHR | aryl hydrocarbon receptor | 196 | F: ATTGTGCCGAGTCCCATATC  R: AAGCAGGCGTGCATTAGACT | 116 |
| CYP1A1 | cytochrome P450 family 1 subfamily A member 1 | 1543 | F: CTTGGACCTCTTTGGAGCTG  R: CGAAGGAAGAGTGTCGGAAG | 212 |
| ABCG2 | ATP binding cassette subfamily G member 2 | 9429 | F: CAGGTGGAGGCAAATCTTCGT  R: ACCCTGTTAATCCGTTCGTTTT | 247 |
| c-Myc | v-myc avian myelocytomatosis viral oncogene homolog | 4609 | F: ATTCTCTGCTCTCCTCGACG  R: CTGTGAGGAGGTTTGCTGTG | 217 |
| KLF4 | Kruppel-like factor 4 | 9314 | F: AGTTCCCATCTCAAGGCACA  R: GCCTCTTCATGTGTAAGGCG | 205 |
| ALDH1A1 | aldehyde dehydrogenase 1 family member A1 | 216 | F: GCACGCCAGACTTACCTGTC  R: CCTCCTCAGTTGCAGGATTAAAG | 129 |
| LGR6 | leucine-rich repeat containing G protein-coupled receptor 6 | 59352 | F: GGATGTGTGCCAGCTTCTTC  R: TGTGGCTTTGAGTCCTCCAT | 172 |
| CXCR4 | C-X-C motif chemokine receptor 4 | 7852 | F: ACTACACCGAGGAAATGGGCT  R: CCCACAATGCCAGTTAAGAAGA | 133 |

**Table S2 Primers for ChIP enrichment detection at selected regions as indicated genes**

| Name | Sequence | Product size(bp) |
| --- | --- | --- |
| ABCG2 | F: CAAGAGCAGGCAGGAAGGA  R: GCAGAGACAGTGGTAATACGAA | 128 |
| c-Myc | F: TACGGAGGAGCAGCAGAGA  R: GGCGGAGATTAGCGAGAGAG | 248 |
| KLF4 | F: GGAGAGTGCGTGGCTTGAA  R: GTCTCGAACACCTGACCTCAA | 187 |
| ALDH1A1 | F: AGAACCAAATTGCTGAGCCA  R: CAAACCCGAGTCAAAGCAGA | 155 |
| LGR6 | F: TTTGCCTCTTGGGTCTCAGTT  R: GACAGCCACGAGGTCACAT | 120 |

Table S3 Antibodies for immune-fluorescence (IF) /histochemistry (IHC), WB or ChIP analyses

| Antigen | Company | Cat#/Species | Dilution |
| --- | --- | --- | --- |
| α-Tubulin | Santa cruz | sc-5286/Mouse | 1:3000 WB |
| β-actin | Sigma | A5441/Mouse | 1:4000 WB |
| Histone 3 | Cell Signaling technology | 9715S/Rabbit | 1:2000 WB |
| AhR | abcam | ab2769/Mouse | 1:200 IHC, 1:300 IF |
| AhR | Santa cruz | sc-5579x/Rabbit | 1:2000 WB, ChIP |
| IKKα | Cell Signaling technology | 11930P/Mouse | 1:1000 WB, ChIP |
| IKKα | Santa cruz | sc-7182/Rabbit | 1:100 IF |
| ABCG2 | Millipore | MAB4146/Mouse | 1:4 IHC; 1:300 WB |
| c-Myc | Cell Signaling technology | 5605/Rabbit | 1:1000 WB, ChIP |
| KLF4 | Cell Signaling technology | 4038/Rabbit | 1:500 WB |
| KLF4 | Santa cruz | sc-20691/Rabbit | 1:100 IHC |
| ALDH1A1 | abcam | ab52492/Rabbit | 1:100 IHC, 1:1000 WB |
| CXCR4 | Millipore | AB1846/Rabbit | 1:100 IHC, 1:1000 WB |
| LGR6 | epitomics | 5540-1/Rabbit | 1:100 IHC, 1:1000 WB |
| CD338-Percp-Cy5.5 | BD Biosciences | 561460 | 5μl/1*10^6cell |
| CD326-BV510 | BD Biosciences | 563181 | 5μl/1*10^6cell |
| Alex Flour^TM^ 488 donkey anti-rabbit | invitrogen | A21206 | 1:100 |
| Alex Flour^TM^ 595 donkey anti-mouse | invitrogen | A21203 | 1:100 |

**Supplementary Material and Methods**

**Western blot analysis and Co- Immunoprecipitation (Co-IP) assay**

Western blot analysis was performed using whole-cell lysates and primary antibodies and a HRP-linked, species-specific anti-rabbit, anti-mouse, or anti-rat IgG (Dallas, Texas, USA). Equal protein sample loading was monitored by hybridizing the same membrane filter with an anti α-tubulin or β-actin antibody (see Table below for detailed antibody information). Proteins were detected with the BIO-RAD ChemiDoc^TM^ XRS+ system. Western blot results were quantitated using ImageJ software and protein expression was normalized to α-Tubulin or β-Actin.

Details of western blot analysis were described previously ^1^. Cells were harvested, washed twice with ice-cold phosphate-buffered saline (PBS), lysed in RIPA buffer and centrifuged at 15,000 × g for 10 min after sonication. The supernatants were collected as whole cell lysates. A quantity of 50 μg of total protein was used for Western blot analysis. For immunoprecipitation experiments, cells were plated overnight in 100 mm^2^ dishes (1.5×10^6^/dish). A quantity of 1 mg of protein was mixed with 40 μl of Protein A-Sepharose beads (Sigma) in the immunoprecipitation assay buffer (1× PBS, 0.5% Nonidet P-40, 0.5% sodium deoxycholate and 0.1% SDS), incubated at 4°C for 2 h with gentle agitation and centrifuged for 10 min at 2000 rpm for preclearing. The recovered supernatant was incubated with 2 μg of anti-AhR (Sigma) in the presence of 1× protease inhibitors at 4°C overnight with mild shaking. The precipitated protein complex was recovered by a brief centrifugation followed by three washes with the immunoprecipitation assay buffer. The harvested beads were re-suspended in 30 μl of 2× SDS PAGE sample buffer and boiled for 5 min to release the bound protein. A 20 μg aliquot of cell lysate was used as an input control. The samples were analyzed by Western blot. The antibodies used for Western blot detection were the AhR, and IKKα antibodies.

**Tissue samples and Immunohistochemistry (IHC) analysis**

Eighteen paired NSCLC and adjacent non-tumor lung tissues were obtained from patients who underwent surgery at the second hospital of Xiangya (Hunan, China) during 2010-2014 and were diagnosed with NSCLC (stage I, II, and III) based on histopathological evaluation. Clinical characteristics including tumor-node-metastasis (TNM) stage were collected. No local or systemic treatment was conducted in these patients before surgery. All collected tissue samples were immediately snap-frozen in liquid nitrogen. The Research Ethics Committee of the second Xiangya hospital approved the study. Written informed consent was obtained from all patients.

One hundred seventy-six NPC patients who were treated by curative-intent radiotherapy (a total dose of 60–70 Gy) using a modified linear accelerator in the Xiangya Hospital of Central South University, China from August 2013 to July 2014 were reviewed. Among these patient, 44 NPC patients without distant metastasis (M0; WHO staging II and III) at the time of diagnosis, comprising 22 radioresistant and 20 radiosensitive patients, were recruited in this study. Radioresistant NPC patients were defined as ones with persistent disease (incomplete regression of tumor) at >6 weeks after completion of radiotherapy or ones with recurrent disease at the nasopharynx and/or neck nodes at >2 months after completion of radiotherapy. Radiosensitive NPC patients were defined as ones without the local residual lesions at >6 weeks or recurrence at >2 months after completion of radiotherapy. Distant metastasis was excluded by skeletal, thoracic, and upper abdominal imaging before radiotherapy. NPC tissue biopsies from these 44 patients were obtained at the time of diagnosis before any therapy with an informed consent and were used for immunohistochemical staining. This study was approved by the ethics committee of Xiangya School of Medicine, Central South University, China.

Nasopharyngeal and related diseases biopsies, validated by pathologist Dr. Desheng Xiao (Xiangya Hospital), were obtained from Pathology Department of Xiangya Hospital. The NPC tissue array was purchased from Pantomics (Richmond, CA, USA). IHC analysis of paraffin sections from NPC tissues or xenograft samples was described previously ^2^. The sections were incubated with antibodies as indicated. The images were surveyed and captured using a CX41 microscope (OLYMPUS, Tokyo, Japan) with the Microscope Digital Camera System DP-72 (OLYMPUS, Tokyo, Japan) and differentially quantified by two pathologists who were from the Second Xiangya Hospital, Changsha, China.

AhR staining was considered positively by ascertaining cytoplasmic and nuclear expression. The determination result was obtained from semi-quantitative classification according to 10 more visual fields (×200). The slides were first scored as 0 (negative), 1 (buff), 2 (pale brown), and 3 (tan). Positive expression of AhR were scored as 0 (negative), 1+ (<10% of positively-staining tumor cells), 2+ (11-50% of positively-staining tumor cells), 3+ (50-75% of positively-staining tumor cells), and 4+ (>75% of positively-staining tumor cells. Both the scores by multiply were regarded as the determination result.

**Quantitative real-time PCR and RNA sequencing**

Cells were harvested with Trizol (Invitrogen). cDNAs were synthesized with SuperScript III (Invitrogen) according to the manufacturer’s protocol. Real-time PCR analysis was performed using the Applied Biosystems 7500 Real-Time PCR System, according to the manufacturer’s instructions. The reactions were performed in triplicates for three independent experiments: the results were normalized to **β**-actin. The primer sequences used were used in the supplementary Table S1. The mean± SD of three independent experiments was shown.

For RNA sequencing, the total RNA samples are first treated with DNase I to degrade any possible DNA contamination. Then the mRNA is enriched by using the oligo(dT) magnetic beads. Mixed with the fragmentation buffer, the mRNA is fragmented into short fragments. Then the first strand of cDNA is synthesized by using random hexamer-primer. Buffer, dNTPs, RNase H and DNA polymerase I are added to synthesize the second strand. The double strand cDNA is purified with magnetic beads. End reparation and 3’-end single nucleotide A (adenine) addition is then performed. Finally, sequencing adaptors are ligated to the fragments. The fragments are enriched by PCR amplification. During the QC step, Agilent 2100 Bioanaylzer and ABI StepOnePlus Real-Time PCR System are used to qualify and quantify of the sample library. The library products are ready for sequencing via Illumina HiSeqTM 2000. The whole RNA-sequencing process and data analysis was conducted by BGI Tech, Shenzhen, China.

**Healthy donor and clinical blood specimens, PBMC preparation from human blood sample, blood spiking experimetns and cell sorting from patients’ blood**

All healthy donor and patient blood specimens were acquired and handled according to the protocols approved by the Second Xiangya Hospital Institutional Review Board. Whole blood from healthy donors, who were not taking medications including those known to affect platelet functions (e.g., nonsteroidal anti-inflammatory drugs or aspirin) within 48 h prior to phlebotomy, was obtained at Second Xiangya Hospital. Whole blood from a total of 10 cancer patients who were receiving care at the Second Xiangya Hospital was obtained in one or more occasions. All blood was drawn into the anticoagulation tubes containing EDTA. Blood specimens were held at RT (20–25 °C) and were processed as soon as possible. For the majority of the experiments the blood was used within 2 h, including transport time from the clinic. Blood specimens that were older than 2 h were discarded. As such, the term “fresh blood” refers to blood used within 2 h.

Mononuclear cells (PBMC) were isolated by density centrifugation on Ficoll- paque PLUS. Whole blood was diluted in an equal volume of phosphate-buffered saline (PBS), then carefully overlaid on Ficoll–Paque PLUS (GE healthcare Bio-Sciences AB, Uppsala Sweden), and centrifuged at 400 g for 30 min at room temperature in a swinging-bucket rotor without the brake applied. The PBMC rich interface was harvested, washed twice in PBS, counted on a cell counter (AcT20, Beckman Coulter, Miami, FL), and adjusted to 1🞨10^7^/ml in PBS.

A549-IR cells were used as spiked cancer cells to simulate cancer samples as a validation test. 10 to 1000 A549-IR cells were mixed with 3🞨10^6^ PBMC in 300 μl volume of PBS. The mixed cells were then added to tubes containing cocktails of fulorochrome-labeled mAbs (5μl of CD45-BB515, 5μl of EpCAM-APC, 5μl of CD338-PerCP-Cy5.5, 1μl of Hoechst 33342, 5μl of DPBS with 2% FBS), mixed and incubated on ice for 30 min, shielded from light. The mixture was washed by FACS buffer for once and went through a FACS Aria II (BD Biosciences, San Jose, CA).

10 ml blood samples were collected from clinic NSCLC patients. PBMCs were prepared from each sample and adjusted to 1🞨10^7^ cells/ml PBS. 3🞨10^6^ PBMC cells were incubated with mixed antibodies, as above described. Cells were gone through the FACS Aria II. The Hoechst 33342-CD45-EpCAM+CD338+ cells were gated the same way as A549-IR cells and sorted into 1640 with 5% FBS for further experiments.

**Targeted GC–MS, 2HG and TCA metabolite measurements**

GC/MS assays were essentially performed as described ^3^. To measure whole-cell associated metabolites, media was aspirated and cells were harvested as described above. Metabolite extraction was accomplished by adding a 10× volume (m/v ratio) of -80 °C methanol:water mix (80%:20%) to the tumor tissue (approximately 100 mg) followed by 30 s homogenization at 4 °C. These chilled, methanol extracted homogenized tissues were then centrifuged at 14,000 r.p.m. for 30 min to sediment the cellular and tissue debris and the cleared tissue supernatants were stored at -80 °C. Two different gas chromatography (GC) separation methods were used, each coupled by negative electrospray ionization (ESI, 23.0 kV) to triple-quadrupole mass spectrometers operating in multiple reaction monitoring (MRM) mode, with MS parameters optimized on infused metabolite standard solutions. In both methods, metabolites were separated by reversed-phase chromatography using 10 mM tributylamine as an ion pairing agent in the aqueous mobile phase. The first method allowed resolution of TCA metabolites: t50, 50% B; t55, 95% B; t57, 95% B; t58, 0% B, where B refers to an organic mobile phase of 100% methanol. The second method was specific for 2HG, running a fast linear gradient from 50% to 95% B (buffers as defined above) over 5 min. In both methods, the column was a Synergi Hydro-RP, 100mm32 mm, 2.1 mm particle size (Phenomenex). Metabolites were quantified by comparison of peak areas with pure metabolite standards at known concentration.

.

References:

1. Shi Y, Tao Y, Jiang Y, Xu Y, Yan B, Chen X*, et al.* Nuclear epidermal growth factor receptor interacts with transcriptional intermediary factor 2 to activate cyclin D1 gene expression triggered by the oncoprotein latent membrane protein 1. *Carcinogenesis* 2012, **33**(8)**:** 1468-1478.

2. He X, Yan B, Liu S, Jia J, Lai W, Xin X*, et al.* Chromatin Remodeling Factor LSH Drives Cancer Progression by Suppressing the Activity of Fumarate Hydratase. *Cancer research* 2016, **76**(19)**:** 5743-5755.

3. Dang L, White DW, Gross S, Bennett BD, Bittinger MA, Driggers EM*, et al.* Cancer-associated IDH1 mutations produce 2-hydroxyglutarate. *Nature* 2009, **462**(7274)**:** 739-744.
